# Recent progress in targeted delivery vectors based on biomimetic nanoparticles

**DOI:** 10.1038/s41392-021-00631-2

**Published:** 2021-06-07

**Authors:** Li Chen, Weiqi Hong, Wenyan Ren, Ting Xu, Zhiyong Qian, Zhiyao He

**Affiliations:** 1 Department of Pharmacy, State Key Laboratory of Biotherapy and Cancer Center, National Clinical Research Center for Geriatrics, West China Hospital, Sichuan University, Chengdu, Sichuan China; 2 Key Laboratory of Drug-Targeting and Drug Delivery System of the Education Ministry, Sichuan Engineering Laboratory for Plant-Sourced Drug and Sichuan Research Center for Drug Precision Industrial Technology, West China School of Pharmacy, Sichuan University, Chengdu, Sichuan China

**Keywords:** Biomimetics, Drug delivery

## Abstract

Over the past decades, great interest has been given to biomimetic nanoparticles (BNPs) since the rise of targeted drug delivery systems and biomimetic nanotechnology. Biological vectors including cell membranes, extracellular vesicles (EVs), and viruses are considered promising candidates for targeted delivery owing to their biocompatibility and biodegradability. BNPs, the integration of biological vectors and functional agents, are anticipated to load cargos or camouflage synthetic nanoparticles to achieve targeted delivery. Despite their excellent intrinsic properties, natural vectors are deliberately modified to endow multiple functions such as good permeability, improved loading capability, and high specificity. Through structural modification and transformation of the vectors, they are pervasively utilized as more effective vehicles that can deliver contrast agents, chemotherapy drugs, nucleic acids, and genes to target sites for refractory disease therapy. This review summarizes recent advances in targeted delivery vectors based on cell membranes, EVs, and viruses, highlighting the potential applications of BNPs in the fields of biomedical imaging and therapy industry, as well as discussing the possibility of clinical translation and exploitation trend of these BNPs.

## Introduction

Even if the emergence of nanotechnology has posted alternative therapeutic opportunities, new delivery challenges appear timely with the participation of nanoparticles. As nanoparticles travel in the bloodstream from the injection site to the target tissue, they will encounter various physiological and cellular barriers that are intrinsically skilled in recognizing and eliminating foreign substances. Multifarious protein-based and cellular constituents and reticuloendothelial system (RES) can interact with the nanoparticles, leading to compromise performance^1,2^. To extend blood circulation, the surface of nanoparticles is generally modified with synthetic polymers such as polyethylene glycol (PEG) or poly(lactic-co-glycolic acid) (PLGA)^3,4^. Plentiful targeting ligands, including aptamers, polypeptides, antibodies, and small molecules, are further grafted to selectively bind only to the target sites while avoiding non-specific interactions with healthy tissues^5^. However, the development of synthetic nanoparticles is impeded by unexpected biological events and material attributes^6–8^. Optimizing on synthetic nanoparticles has always been implemented for effectively targeted delivery with minimal side-effects^9,10^. Thus, tremendous efforts have recently been devoted towards bioinspired nanotechnology, where design cues for effective delivery are taken from the organism^11,12^.

Biological vectors, nano-/micro-sized particles extracted from the organism, consist of endogenous membrane debris such as cell membranes, extracellular vesicles (EVs), and exogenous substances such as viruses^13^. Among the various vectors (Table 1), cell membranes are mainly derived from the cancer cell^14^, neutrophils^15^, natural killer (NK) cells^16^, macrophages^17^, erythrocytes^18^, and platelets^19^. As the functional limitation of single-cell type, the hybrid cell membranes incorporated with multi-functions of several cell types are also involved in this category^20^. Except for the integral cell membrane, the cell can also secrete EVs, principally divided into exosome (Exo) and microvesicles (MVs)^21^. The viral vectors are acquired from mammalian virus, bacteriophage, and plant virus according to different hosts^22^. These biological vectors inherit structural and functional complexity from original donors, serving as native substances to reduce undesired immune response and avoid being cleared straightly^23^.

**Table 1:** Currently explored natural vectors for nanoparticles camouflage

Vectors		Advantages	Defects	References
Cell membrane vectors	Red blood cell membrane	Prolonged blood circulation; high biocompatibility; immune evasion	Lack of specific targeting ligands on red blood cell membranes	^12,40,314^
	Cancer cell membrane	Homotypic targeting capability; elicit specific immune response; easy to culture in vitro on a large scale	Relatively short circulation time	^20,309^
	Immune cell membrane	Targeting to the inflammatory site; immune evasion; elicit specific immune response	The least component in the blood; only effective to certain tumors	^87–89^
	Hybrid cell membrane	Multifunctional integration of individual cell types	Lack of productive technique	^20^
Evs vectors	Exosome	Tiny diameter; reduce phagocytosis; extravasate through tumor vessels; across biological barriers	Limited to obtain high yields of pure Exo	^21^
	Microvesicle	Inherit tumor-targeting capability	More heterogeneous in size	^178^
Viral vectors	Mammalian viral nanoparticles	Traditionally used in gene delivery strategies	Causing horizontal genetic transfer events; stimulate undesirable immune responses	^315^
	Bacteriophage-viral nanoparticles	Non-infectious to mammals; do not undergo alterations in their natural tropism or mutation; reduce side-effects and enhance bacterial targeting	Problematic in the repeated application of a therapeutic cargo; rapidly cleaned by the host RES	^238,316^
	Plant virus-viral nanoparticles	Non-infectious to mammals	Do not exhibit tissue tropisms	^22^

**Fig. 1:**
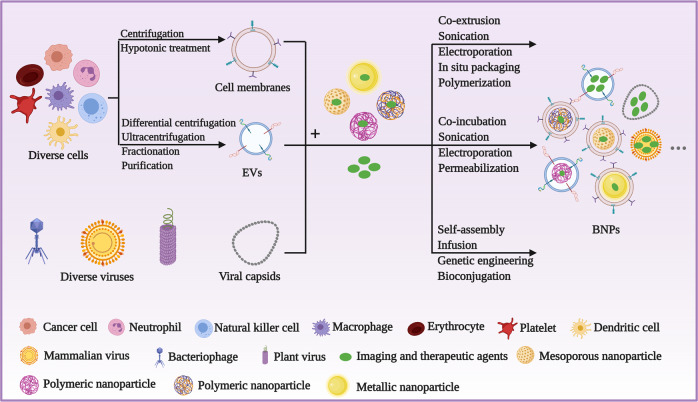
Schematic depicting the typical procedures to construct various BNPs. Both cell membranes and EVs derived from diverse cell types can be obtained, whereas diverse viruses and their capsids serve as vectors directly or indirectly. These biological vectors interact with drug-loaded synthetic nanoparticles or functional agents to form BNPs via multiple methods

Biomimetic nanotechnology takes advantage of these biological vectors to camouflage drug-loaded synthetic nanoparticles or carries functional agents for indirect or direct targeted delivery (Fig. 1). The functionalization of synthetic nanoparticles with natural cells or viral nanoparticles (VNPs) mainly consists of three steps, isolation of biological vectors, obtaining functional agents, and ultimately camouflaging the functional agents with the biological vector. With the same source cells, the isolation process of membrane vesicles from source cells is roughly uniform, including cell isolation, cell lysis, centrifugation, fractionation, purification, etc^24,25^. Different from vesicles’ isolation, viral vectors can be obtained by removing genetic material of viruses or self-assembly of repeating protein subunits^22^ Then, the vesicles and viral vectors are mainly fabricated to form multiple biomimetic nanoparticles (BNPs) by the following methods: co-extrusion, sonication, electroporation, polymerization, self-assembly, infusion, genetic engineering, and bioconjugation^20,22,26^ Notably, the whole process is supposed to proceed as mildly as possible at a low temperature to retain the bioactivity of BNPs^27^.

**Fig. 2:**
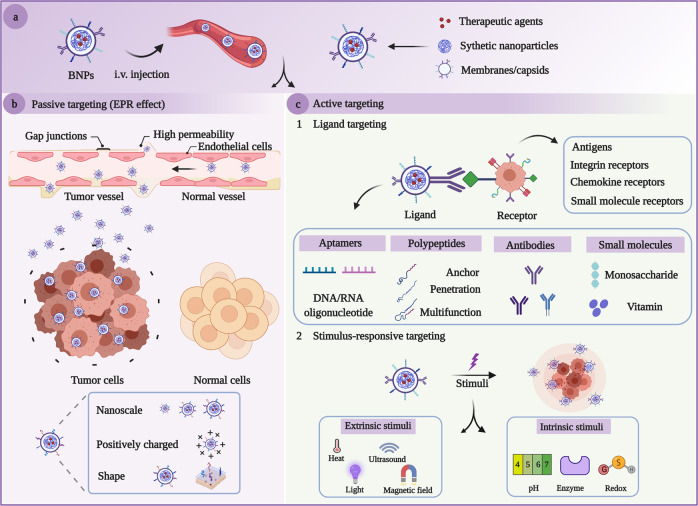
Schematic mechanisms of targeted delivery based on BNPs. **a** BNPs loaded with drugs were injected into the organism intravenously. **b** The passive targeting of BNPs towards tumors through the EPR effect. As the leaky vasculature exists in tumor vessels, BNPs are allowed to pass through the pathophysiological walls rather than regular walls, leading to the accumulation of BNPs within the tumor due to the characteristic size, shape, surface charge of the nanoparticles. **c** Active targeting enables uptake of BNPs through the ligand-mediated pathway and stimulus-responsive pathway. Ligand-mediated targeting leverages the high expression of specific receptors on the surface of targeted cells by keeping them engaged with the targeting ligands. In the presence of intrinsic and/or extrinsic stimuli, BNPs attempt to accumulate in microenvironments of disease tissues and realize environment-responsive drug release

Targeting strategies based on BNPs are generally divided into two main categories: passive targeting and active targeting (Fig. 2). Nanoscale biomimetic particles enable prolonged half-lives and contribute to their accumulation in the tumor sites via enhanced penetration and retention (EPR) effect, namely passive targeting. However, passive targeting is mainly dependent on the nanoparticle size as passive diffusion is achieved by the tumor microenvironment. Besides, the camouflaged nanoparticles bestow superior interaction between extraneous nanoparticles and the physiological environment owing to their homologous-targeting ability. As natural vectors have demonstrated definite passive targeting and homologous-targeting ability, there is still significant room for precise and accurate targeting. Except passive targeting and homologous-targeting strategies, natural vectors have been widely modified with various ligands, which we focus on in this review, to achieve active targeting via ligand–receptor interactions. Nanoparticle modification has dynamic influences on transforming the patterns and pathways of nanoparticle interactions with organisms. Adopting an applicable functionalized modification is of great importance to target specific intracellular pathways for superior therapeutic interventions with fewer side-effects^28–30^. Surface modification and genetic engineering are conducted on the vast majority of BNPs, which can be developed as promising vehicles to deliver therapeutic and contrast agents for the imaging, treatment, and prevention of various diseases^31–34^. Mechanistic studies have contributed invaluable insight to prosperous theranostics, yet there are a handful of clinical trials implementing EVs and viral vectors in fields of clinical medicine.

Overall, biological vectors have been extensively developed to deliver payloads. Leveraging the natural properties of biological vectors and surface modification technologies, BNPs are endowed with high specificity, long circulation, low immunogenicity, and biocompatibility. It is expected that biological vectors will serve as a promising candidate for imaging and therapeutics, particularly in the circumstances of demanding high targeting specificity. Hence, this review narrows the focus on biological vectors derived from cell membranes, EVs, and viruses to deliver imaging and therapeutic agents for refractory disease therapy. Finally, we overview the landscape of clinical developments on these BNPs, as well as sketch the challenges and prospects in the future.

## Cell membrane vectors

**Fig. 3:**
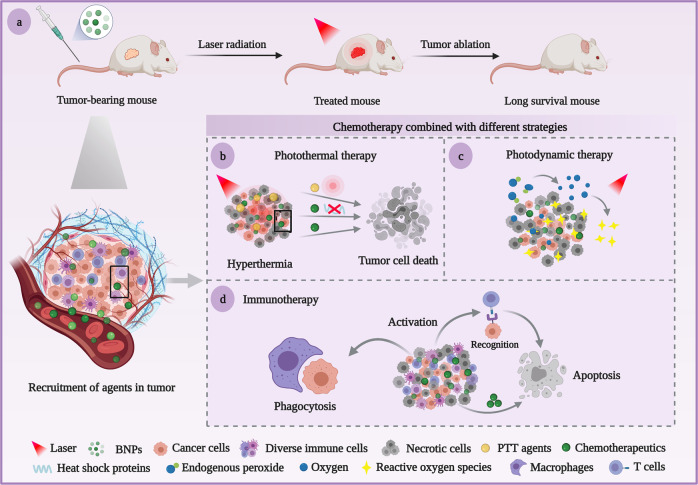
Schematic illustration of chemotherapy combined with different modalities against tumors based on cell membrane vectors. **a** After the tumor-bearing mice being injected with BNPs targeting tumor sites, the tumor is obviously inhibited by combination therapy of chemotherapeutic with laser radiation or immune response. **b** PTT agents elevate the temperature around tumor sites by photothermal conversion, and chemotherapeutic agents synergistically down-regulated the expression of heat shock proteins, reduce the heat resistance, and directly induce apoptosis of tumor cells. **c** Aside from tumor toxicity of chemotherapeutic agents, endogenous peroxide is effectively converted into oxygen to alleviate tumor hypoxia and oxygen alters to tumor-toxic reactive oxygen species under laser radiation for antitumor effect enhancement. **d** The antigens on the surface of BNPs elicit the immune response to tumor cells, such as T-cell activation and macrophages to kill tumor cells

Synthetic nanoparticles cloaked with biological cell membranes for cancer therapy have drawn enormous attention while serving as initial cells that deliver agents to the targeted issues. BNPs generally include nanoparticles camouflaged with cell-derived membranes, consisting of unique membrane substances and effective agents in diverse approaches^35^. Among multiple membrane camouflage strategies, a single-cell membrane optimizes the nanoparticles’ biological function, whereas other bio-functions can be attached to specific applications^20^. Hybrid cell membrane possesses multifunctional membrane properties based on different cell types within an integrated biomimetic system^36^. Cell membrane-coated nanoparticles, the cohesion of biological membrane and nanomaterials, remain both intricate biological functionalities of cell membranes and favorable physicochemical characteristics of synthetic nanoparticles^37^. These BNPs camouflaged by membranes derived from innate cells (mainly mention about tumor cells, erythrocytes, immunocytes) can be delivered to specific lesions, escaping from protein adsorption and phagocytosis of RES to realize prolonged circulation in vivo^37,38^. The BNPs loaded with chemotherapeutics can be integrated with different therapeutic modalities for combinatorial therapy, particularly against malignant tumors (Fig. 3)^39^. This section summarizes the research progress of BNPs based on cell membranes in targeted drug delivery systems (TDDS), which employs various membranes as vehicles for accurate imaging and therapy.

### Red blood cell membrane vectors

Red blood cell (RBC) membranes are natural long-circulating delivery vehicles. The prime cell membrane donors extend nanoparticle residence time owing to many “self-markers” (e.g., CD47 proteins, peptides, glycans, acidic sialyl moieties) preventing being phagocytosed by macrophages in RES to prolong its half-life^40–42^. Mainly, characteristic structure and surface proteins of RBC have been considered as design cues to conceive a more optimal delivery nano-system for tumor-targeting therapy. To optimize the function of RBC membranes, several methods including avidin–biotin interactions-based insertion and lipid insertion technique were leveraged to modified RBC membranes. Thus, RBC membrane-based nanodevices have promise for targeted delivery ranging from chemotherapeutic drugs to detoxification agents.

A theranostic nanoplatform (RMSNs-Dox/Ce6) was constructed to realize blood suspensibility and laser-activated tumor-specific drug release simultaneously via a noninvasive method^43^. Anticancer drug doxorubicin (Dox) and photosensitizer (PS) chlorin e6 (Ce6) were loaded onto mesoporous silica nanoparticles (MSNs), which were subsequently camouflaged by RBC membrane. RMSNs-Dox/Ce6 showed long-time circulation, deep tumor penetration, and precisely controlled drug release performance, significantly inhibiting the primary tumor growth and prevented breast cancer from metastasizing to the lung. Rapamycin (RAP)-loaded PLGA were camouflaged with RBC (RBC/RAP@PLGA) to escape from mononuclear phagocytic system clearance for atherosclerosis treatment^18^. EPR effect was found in atherosclerotic plaque on the basis of leaky endothelium in inflammation and microvessels, allowing the nanoparticles to permeate the vascular wall and accumulate in the pathological lesion^44^. Less macrophage-mediated phagocytosis in the bloodstream and superior accumulation of RBC/RAP@PLGA were observed within the established atherosclerotic plaques of ApoE^−/−^ mice, leading to a targeted drug release. Whereas, the specificity function of RBC is limited for the lack of specific targeting ligands on RBC membranes while coagulation of platelets stimulates tumorigenesis, invasion, and metastasis by different means^45,46^. Receptor-mediated endocytosis is a vital mechanism for drug delivery to glioma, thus some attempts have been dedicated to the development of active targeted RBC nanoparticles that are decorated with specific ligands targeting designated cell receptors. Chai^47^ developed the RBC membrane modified with tumor-targeting peptides c(RGDyK) through a facile insertion method involving avidin–biotin interactions. Then, the RBC membrane-coated drug nano-system was established by encapsulating docetaxel (DTX) nanocrystals. RGD-RBC@DTX demonstrated excellent tumor cell specificity and potent therapeutic effect in both orthotropic glioma and subcutaneous tumor-bearing mice models. Besides, the lipid insertion technique was used to prepare T7/NGR-co-modified RBC membrane-coated solid lipid nanoparticles, enhancing tumor-targeting and therapeutic efficacy over glioma^48^.

RBC-based BNPs have been developed as a potential nanoplatform for intercepting and neutralizing bacterial toxins since RBC membranes with natural affinity to the toxins^49,50^. Ben-Akiva et al.^51^ designed a detoxification device by leveraging RBC membrane-coated anisotropic polymeric nanoparticles (RBC@PLGA). RBC underwent hypotonic lysis and was then sonicated to generate nanoscale vesicles. Spherical, prolate, or oblate ellipsoidal PLGA nanoparticles were camouflaged by processed RBC membranes to evaluate their uptake by macrophages and detoxification activity in vitro and in vivo. Compared with bare PLGA nanoparticles and spherical RBC@PLGA, both prolate RBC@PLGA and oblate ellipsoidal RBC@PLGA were bestowed with the wider surface area owing to their anisotropic shape, exhibiting statistically significant evade elimination and strong detoxification in mice models. An innovative research reported that the PLGA core was clocked with RBC derived from humans to form toxin nano-sponges (hRBC-NS) for broad-spectrum neutralization^52^. hRBC-NS completely neutralized the toxins’ hemolytic activity in both preincubation and competitive settings. Most importantly, toxins inserted in hRBC-NS were followed by inactivation, which observed no toxicity to either cells or animals. hRBC-NS promoted the potential of autologous cells applying to human beings and amplifying feasible antimicrobial prophylactics and therapeutics. However, whether the affinity of cell membrane vectors and inner cores could ensure effective encapsulation remains ambiguous. To address this dilemma, amphotericin B (AmB)-loaded cationic liposomes were anchored with a peptide ligand, which can interact with band 3 intracellular domain on RBC membrane (RBC-LIP-AmB), guaranteeing effective and accurate loading process^53^. RBC-LIP-AmB exhibited a distinct ability to neutralize hemotoxin released by the pathogenic fungi, and protected the mice from *Candida albicans* infection as well as prolonged survival time. Other than the above, RBC membrane can camouflage bovine serum albumin for malignant melanoma metastasis^54^, perfluorocarbon for hypoxia alleviation^55^, ZGGO@mSiO2 for bioimaging and therapy of breast cancer^56^, PAAV-SNO copolymer for immune cold, and reprogramming immunosuppressive tumor therapy^57^.

### Cancer cell membrane vectors

BNPs camouflaged by cancer cell membrane (CCM) are distinguished as endogenous cancer cells. Therapeutic/contrast agents can be positively delivered to the lesion owing to the molecular recognition and adhesion on the surface of cancer cells, and the agent is concentrated enough to be worth carrying out effective imaging and therapy^58^. Homotypic targeted delivery is realized by adopting camouflaged nanoparticles with natural extracts or CCM elements, allowing for the self-recognition with source cancer cells^24,59^. Homotypic adhesion capability depends on diversified membrane proteins including galectin-3^60^, tissue factor-antigen^61^, E-cadherin^62^, where there is a natural synergy between the proteins.

In vivo, optical imaging system is vital for specific cancer imaging, not only for early diagnosis and prognosis, but also the investigation of cancer cell metastasis^63,64^. Among the plentiful nanomaterials that emerged, up-conversion nanoparticles (UCNPs), a kind of state-of-the-art fluorescent probes, have been harnessed previously for tumor imaging by coating them with cell membranes. The modified UCNPs endow the original nanoparticles with stronger optical light penetration, good performance of near-infrared (NIR) fluorescence emission, excellent light resistance, and high specificity and affinity to overcome the limitation of traditional technologies^65^. To execute active tumor targeting, Rao et al.^66^ designed a bioengineered CCM functionalized nanoplatforms based on UCNPs (CC-UCNPs). MDA-MB-435 human breast cancer cells were incubated with UCNPs coated on four different CCM (MDA-MB-435 human breast CCM, DU 145 human prostate CCM, CAL 27 human squamous CCM, and HCT 116 human colorectal CCM) accordingly, and then the cells were analyzed by flow cytometry. MDA-UCNPs showed the strongest Cy5 fluorescence that verified a homologous adhesion between MDA-UCNPs and MDA. Ulteriorly, when BALB/c nude mice bearing MDA-MB-435 breast tumor xenografts were treated with the same dosage injection of different fabricated UCNPs, the obviously bright up-conversion luminescence (UCL) was detected in MDA-UCNPs groups for both in vivo and ex vivo UCL imaging. All the results confirmed that MDA-UCNPs presented immune evasion and homologous-targeting capabilities obtained from the source cancer cells. In terms of up-conversion tumor imaging, UCNPs coated with different types of membranes exhibited remarkable optical characteristics, whereas the penetration depth and signal-to-noise ratio (SNR) of rare-earth-doped nanoparticles (RENPs) are restricted by short-wavelength luminescence^67^. Although RENPs can penetrate ~7 mm in the second near-infrared window (NIR-II), it remains poor tumor imaging efficiency^68^. To overcome this predicament, Zhang et al.^67^ camouflaged PEGylated-RENPs with the CCM (CC-RENPs) to decrease the uptake in the liver and spleen system, and improve tumor imaging performance in NIR-II window. Notably, MDA-MB-231 tumors were distinctly mapped with CC-RENPs at 24 h post injection, which can conduct tumor resection thoroughly by real-time NIR-II imaging. CC-RENPs possess great potential as a safe NIR-II image probe substitute for medical imaging applications in the future. In addition to NIR dyes that are often wrapped in the cores for better imaging effects, noble metal nanoparticles coated with CCM can also assist in imaging. As published recently by Xie et al.^69^, gold nanoparticles were cloaked with CCM to elucidate the molecular mechanism of CCM-coated nanoparticles for homotypic targeting. The findings suggested that integrin α_v_β_3_, a cell surface receptor overexpressed in cancer cells, is vital for the cell-specific recognition of CCM-coated nanoparticles.

CCM-camouflaged nanoparticles can integrate membrane binding to immunoadjuvant and tumor-associated antigens, thus facilitating antitumor immune responses by effective delivery to specific antigen-presentation cells^70,71^. Besides, as the camouflaged nanoparticles externally share the same adhesion molecules with source cells, they present the ability to target the source cells specifically, which accounts for the mechanism of homotypic targeted delivery applied in cancer therapy^37^. For the sake of stimulating a strong cellular immune response, a perfect cancer vaccine is supposed to compose both effective immunogenic cancer-specific antigens and potentiating adjuvant^72^. Tumor cell membrane proteins are deemed as tumor antigens and inorganic salts as immunoadjuvants, inducing specific cellular immunity and humoral immunity to regulate immune function for prophylaxis and therapy^73^. Jiang et al.^74^ constructed a biomimetic artificial antigen-presenting cells (APC) nano-system (CD80/OVA NP), directly activating T cells to fight against cancer in the absence of natural APC. To achieve this goal, the membranes originated from cancer cells presenting intrinsic peptide epitopes were genetically engineered to express a co-stimulatory signal CD80, which triggered tumor antigen-specific immunological reaction. B16-OVA tumor-bearing mice subcutaneously injected with CD80/OVA NP exhibited smaller average tumor sizes, longer survivals, and more desirable prophylactic efficacy. Another potential vaccine delivery system was produced by aluminum phosphate-CpG nanoparticles camouflaged with B16F10 tumor cell membranes (APMC)^14^. With the subcutaneous injection of APMC in mice, APMC were significantly drained to lymph nodes, which permitted the co-uptake of tumor antigens and CpG by lymph node-resident APC, facilitated maturation of the cells, and enhanced lysosomal escape of APMC. In preventive and therapeutic melanoma models, the induced responses dramatically inhibited tumor growth and prolonged lifespan in mice, providing great hopes for precision vaccine and clinical application. Similarly, calcium pyrophosphate nanoparticles were fabricated with lipids and B16-OVA tumor cell membranes to develop a biomimetic antitumor nano-vaccine that demonstrated admirable tumor therapeutic and prophylactic effects^75^. Except for leveraging antigens, Yang et al.^72^ designed an effective anticancer vaccine (NP-R@M-M) that the mannose-modified CCM was harnessed to camouflage exogenous adjuvant (imiquimod)-loaded PLGA nanoparticles. With excellent performance to inhibit tumor growth as a preventive vaccine, the synergy of NP-R@M-M and checkpoint-blockade therapy further proved distinguished therapeutic efficacy to suppress B16-OVA tumors.

In recent years, attractive BNPs with multifunctionality motivated by photodynamic-, photothermal-, or pH response provide a bright future for targeted delivery therapy^76^. Oxygen-dependent photodynamic therapy (PDT) is relatively limited for the therapy of solid tumors with hypoxia^77^. To get over the obstacle, Wang et al.^78^ proposed a novel nanotherapeutic agent (Au@Rh-ICG-CM) that integrated the advantages of hypoxia regulation, tumor accumulation, bimodal imaging, and mild photothermal effect into one single nanoplatform. Au@Rh dramatically catalyzed oxygen generation from endogenous hydrogen peroxide and indocyanine green (ICG) as PS transforming oxygen to tumor-toxic ^1^O_2_. Camouflaging ICG-loaded Au@Rh nanostructures with CCM enables tumor targeting via homologous binding to MDA-MB-231 tumors, selectively accumulating in the tumors to produce tumor-toxic ^1^O_2_ and enhancing photodynamic therapeutic efficiency. Inspired by core-shell nanostructure, a multifunctional biomimetic system (ICNPs) made of ICG and CCM was designed to combine cancer-targeted imaging and photothermal therapy (PTT)^79^. Given that the functionalization of the homologous binding adhesion molecules in CCM, ICNPs facilitated endocytosis and accumulation of homologous-targeting tumor in vivo. Through NIR-FL/PA dual-modal imaging, ICNPs could monitor dynamic distribution in real-time, maintaining high spatial resolution and strong penetration. Simultaneously, ICNPs exerted a significant photothermal effect to eliminate xenograft tumors completely following irradiation with NIR light. Sun et al.^80^` harnessed CCM to camouflage Dox-loaded gold nanoparticles with hyperthermia-triggered drug release and homotypic target for inhibition growth and metastasis of breast cancer Unlike normal three-dimensional (3D) nanosphere, CCM was integrated with in situ synthesized Au nanostars to deliver Dox (NS-M@Dox)^81^. NS-M@Dox maintained a quasi-2D flattened morphology on account of NS enhancing the rigidity of CCM. Compared with sphere nanoparticles, the flattened morphology increased the potential interacting area between the nanoplatform and extracellular surface of targeted cells, which demonstrated more obvious homotypic tumor-targeting efficacy under NIR laser.

Homotypic membrane mutual recognition is one of the most potential and valid strategies for glioblastoma multiforme (GBM). Earlier on, Dox-loaded magnetic iron oxide nanoparticles were camouflaged with CCM derived from UM-SSC-7 and HeLa, respectively, in order to verify preferential cancer cell self-recognition and tumor self-targeting ability of CCM^82^. The nanoplatform exhibited strong potency for tumor treatment in vivo and magnetic resonance imaging (MRI). Owing to the homotypic recognition of GBM cells, Pasquale’s group exhibited a novel drug delivery system with enhanced targeting features that Dox was loaded in boron nitride nanotubes after which coated with GBM cell membranes (Dox-CM-BNNTs)^83^. In both in vitro multi-cellular culture and dynamic model, Dox-CM-BNNTs presented significant homotypic targeting and selective death in U87 MG cells while no obvious side-effect showed in normal cells. The excellent internalization efficiency of U87 MG cells can be explained by GBM cell membranes surface protein recognition that triggers mutual interactions in homotypic membranes through multiple internalization pathways^83,84^. In another research, Liu et al.^85^ successfully constructed a core-shell nanostructure for prostate cancer (PC) therapy where MSNs were leveraged as the core to load Dox, CaCO_3_ interlayer as sheddable pH-sensitive gatekeepers to sustain drug release, and LNCaP-AI prostate CCM as the outermost layer (shorten as Dox/MSNs@CaCO_3_@CM) to enhance tumoritropic accumulation. Different from regular cancer cell camouflaged nanoparticles, Dox/MSNs@CaCO_3_@CM with a unique layer enabled an acidic tumor microenvironment to control drug release. In vivo and in vitro studies manifested that Dox/MSNs@CaCO_3_@CM could distinctly promote apoptosis-associated death in prostate carcinoma cells and obviously curb tumor growth. CCM-camouflaged nanoparticles can benefit from a unique structure that allows them to avoid being cleared by the immune system, regulate immune response, and prompt them to accumulate at specific focal sites^27^. In addition, CCM originated from head and neck squamous cell carcinoma was developed to camouflage cisplatin-load gelatin nanoparticles for personalized therapy in patient-derived xenograft models^86^. Significant targeting capability and complete tumor elimination were observed with the match between the donor and host. TDDS based on membranes derived from various cancer cells can open up an exciting frontier to exploit imaging agents, chemotherapeutics, anticancer vaccines, and adjuvants with combination therapies to enhance the therapeutic effect.

### Immune cell membrane vectors

Immune cells, including neutrophils, NK cells, macrophages, and etc., are capable of mounting an active immune response to elicit inflammation, tumor targeting, suppression of tumor metastasis on account of the specific immune functions of cell membrane-bound complex proteins^87–89^. Camouflaging naive cell membranes onto synthetic nanoparticles, BNPs inherit the antigenic profile from source cells and serve as decoys that neutralize heterogeneous and complex pathological constituents, regardless of their structural specificity^49^. Recent studies related to malignant tumor and inflammatory diseases demonstrated that BNPs made up of using immune cell membrane vectors along with synthetic nanoparticles hold immense promise for drug-targeted delivery.

#### Neutrophil membrane vectors

Given that the tropism of neutrophils migrating to inflammatory tissue and engagement in multifarious inflammatory responses, activated neutrophils are deemed as targeted drug delivery vectors for the therapy of inflammation-related diseases, including malignant tumors, autoimmune conditions, and cardiovascular disease^90,91^. Neutrophil membranes were employed to camouflage onto sparfloxacin-loaded polycaprolactonepoly(ethylene glycol) nanoparticles (NM-NP-SPX) to cure lung inflammation^15^. Antibiotic drugs SPX could be precisely delivered to the sites of inflammatory lungs accurately, extending the half-life of SPX, and minimizing the cytotoxicity against normal tissues. Zhang et al.^87^. proposed neutrophil membrane-coated PLGA nanoparticles with broad-spectrum anti-inflammatory effect for rheumatoid arthritis intervention. The BNPs inherited the antigenic exterior and membrane functions of the original cells, which enabled them to be ideal decoys of neutrophil-targeted biological molecules. The nanoparticles neutralized proinflammatory cytokines, suppressed synovial inflammation, and alleviated joint damage in inflammatory arthritis via dampening the inflammation cascade. In one recent study, methotrexate (MTX)-loaded cationic liposome was encapsulated in neutrophil without removal of endocyte (MTX-liposome/neutrophils) for targeted delivery to inflammatory sites^92^. The oriented accumulation of MTX-liposome/neutrophils was demonstrated in both inflamed skeletal muscle and myocardial ischemia–reperfusion injury mice model, exhibiting the potential to regenerate inflamed tissue.

Two relevant studies demonstrated that the combination therapy based on neutrophile vectors and paclitaxel (PTX) had a synergetic effect on the malignant tumor. PEGylated gold nanorods served as photothermal agents and PTX-loaded neutrophils as inflammation-mediated active targeting chemotherapeutic agent^93^. Though PTT may promote tumor regeneration, neutrophils transformed the demerits of PTT-induced inflammation into merits, showing a strong suppression effect against liver cancer. The other study combined neoadjuvant chemotherapy with local radiotherapy for effective gastric cancer treatment^94^. Notably, researchers harnessed human neutrophils to load Abraxane to form cytopharmaceuticals, which inhibited tumor growth superiorly with the local radiation. Apart from this, neutrophils can facilitate metastatic initiation of breast cancer cells in lung colonization, and the connection between neutrophils and circulating tumor cells (CTCs) prompts the process of the cell cycle in the blood and tends to metastasis^95,96^. Distant metastases of tumors are rooted in the invasion, dissemination, and colonization of CTCs. Thereby, they were assumed to be favorable diagnostic biomarkers and therapeutic targets for restraining metastasis^97^. Previous research findings laid a solid foundation to focus on the interaction between neutrophils and CTCs for targeted inhibition of breast cancer cell metastasis. Kang’s group conceived the TDDS that camouflaged the surface of carfilzomib-loaded poly(latic-co-glycolic acid) nanoparticles with neutrophils membranes (NM-NP-CFZ)^98^. Through intravenous administration in mice, the abnormal expression of S100A9 in the lungs was more significantly reversed and decreased by 71% compared with NP-CFZ (reduced by 44%) and CFZ (reduced by 41%), and NM-NP-CFZ-induced metastasis was reduced by 87.2% after treatment. NM-NP-CFZ possessed the preferable capability to optionally eliminate CTCs in the bloodstream and suppress the emergence of early metastasis. Thus, NM-NP-CFZ was considered to be promising BNPs for inhibiting whole-stage metastasis and treatment applying to various cancers.

#### NK cell membrane vectors

With extensive adoption of NK cells in tumor immunotherapy, the desirable nanostructure camouflaged NK cell membranes always tend to realize targeted delivery with biocompatibility for cancer immunotherapy^99^. NK cells can directly target tumor cells through inhibition and activation of receptors on the surface, accordingly killing the tumor cells without prior sensitization^100^. Activated NK cell membranes with receptors were extracted from NK-92 cells and extruded with fused Dox-loaded liposomes (Dox@NKsomes) to construct BNPs for targeted drug delivery^23^. Treatment effectiveness evaluation about Dox@NKsomes was carried out on the solid tumor induced by human breast cancer MCF-7 cells. Compared with bulk Dox, the tumor inhibition rate of Dox@NKsomes was increased by 14.81%, which remarkably elicited antitumor activity and exhibited potential tumor-targeted cancer therapy ability. The tumor-homing ability was ascribed to the tendency of NK-92 cells to selectively accumulate in the tumor microenvironment, which was based mostly on the overexpression of NK cell receptor ligands such as NKG2-D in cancer cells^101,102^. A separate recent study found that primary and abscopal tumor can be suppressed by using a tumor combined therapeutic system, which camouflaged PS 4,4,4′′,4′′′-(porphine-5,10,15,20-tetrayl) tetrakis (benzoic acid)-loaded mPEG-PLGA nanoparticles with NK cell membranes (NK-NPs)^88^. Shotgun proteomics studies elucidated that NK cell membranes enabled NK-NPs to target tumors and facilitate the polarization of proinflammatory M1-macrophages to stimulate antitumor immunity. Under NIR laser radiation, NK-NPs induced apoptosis of tumor cells so as to augment the antitumor immune effect of NK cell membranes. All the results demonstrated that NK-NPs could target tumors, eliminating the primary tumors and preventing pre-existing distal tumor growth. NK cell membrane was also utilized to camouflage PLGA polymeric nanoparticles with MRI contrast agents Gd lipid for tumor-targeted imaging^16^. The engineered BNPs exhibited longer circulation half-life (~9.5 h) and higher tumor accumulation (~10% of injected dose) in MCF-7 induced tumor-bearing nude mice, confirming the tumor affinity of NK cell membrane fabricated biomimetic nanostructure for targeted bioimaging applications.

#### Macrophage membrane vectors

With the exception of neutrophil membranes and NK cell membranes, macrophage membranes can also be harnessed as drug/contrast adjuvant carriers for targeted therapy and pulmonary metastasis inhibition of breast cancer. After separating the membranes from macrophages, the fellows rebuilt the membranes into vehicles and fused the unit onto the fluorescent UCNPs (MM-UCNPs)^103^. MM-UCNPs had an excellent performance of cancer-targeting capability and biocompatibility in BALB/c nude mice bearing MCF-7 breast tumor xenografts. In the process of lung metastasis of breast cancer, the interaction between α4 integrin of macrophages and the vascular cell adhesion molecule-1 (VCAM-1) of cancer cells accelerates the survival of metastatic cells and triggers the generation of metastatic lesions^104,105^. Macrophage membranes were adopted to camouflage emtansine liposome (MEL) for enhancing the ability to target specific metastasis of breast cancer, so as to inhibit the lung metastasis of breast cancer^89^. MEL significantly facilitated intracellular uptake in metastatic 4T1 breast cancer cells and suppressed cellular activity. Consistently, compared with other groups (emtansine, MEL, and blocked MEL), MEL exhibited outstanding ability to specifically target and inhibit lung metastases of breast cancer in the lung metastatic breast cancer model. As another example, Zhao et al.^106^ developed BNPs based on macrophage membranes and quercetin-loaded hollow bismuth selenide nanoparticles (M@BS-QE NPs), which were expected to inhibit lung metastasis of breast cancer in combination with PTT. It is worth noting that M@BS-QE NPs can be recruited by C–C chemokine ligand 2 (CCL2)/CCR2 and actively target to α4/VCAM-1 to achieve superior tumoritropic accumulation. Owing to the high X-ray attenuation coefficient of bismuth selenide nanoparticles, M@BS-QE NPs performed well in both computed tomography (CT) imaging and infrared imaging that can serve as contrast agents and medicines for the intervention of breast cancer. Natural macrophage membranes with associated membrane proteins were reconstructed into vesicles without loss of their inflammatory tumor-homing ability. Contrary to the phagocytosis property of macrophages, macrophage membrane-camouflaged nanoparticles can be swallowed by tumor cells to achieve drug delivery. Once the nanoparticles were accumulating at tumor sites, the outer membrane becomes a barrier hindering drug release. To realize precisely controlled drug release, a macrophage membrane-camouflaged biomimetic delivery system (cskc-PPiP/PTX@Ma) that can improve the uptake of PTX by targeted delivery and controlled drug release depending on the different pH value in the tumor microenvironment^17^. In a mildly acidic extracellular microenvironment (pH = 6.5), the internal PPiP/PTX nanoparticles served as an H^+^-absorbing proton sponge, and the nanoparticles were released from the ruptured coating after equilibrium disruption of the macrophage’s vesicle structure. After internalized by the cancer cells, the lower intracellular pH (pH = 5.0) ultimately resulted in the synthetic nanoparticle disassembly and drug release. Furthermore, macrophage membranes have been widely leveraged as promising vectors for camouflaging functional materials, including MSNs, Au, and iron oxide nanoparticles, extending the retention time of nanoparticles and enhancing the therapeutic effect of cancer with PTT^107–109^.

### Other cell membrane vectors

Owing to natural targeting abilities and biocompatibility, the other cell membranes also have been employed to interact with plenty of different disease-relevant substrates, including acute myocardial infarction, cancer, atherosclerotic plaque, and bacteria^11,34^. Li et al.^110^ designed a biomimetic nano-system (IL1-PMs) that functioned as an interleukin-1 (IL1) decoy reducing the local inflammatory response in the injured heart. Platelet membrane microparticles (PMs) conjugated with anti-IL1β antibody Gevokizumab to neutralize IL1β after acute myocardial infarction and further prevent adverse cardiac remodeling. IL1-PMs exhibited infarct-homing ability in C57BL/6 mice and inhibition of cardiomyocyte apoptosis by neutralization of IL1β. In another recent study, metal-organic nanoparticles were camouflaged by a layer of naturally derived platelet membrane for the effective targeted delivery of siRNA against survivin^19^. The nanoplatform was implemented to significantly silence gene expression and strongly inhibit tumor growth when designed against a therapeutically relevant target in subcutaneous SK-BR-3 tumor-bearing mice. Beyond that, myeloid‑derived suppressor cell (MDSC) membrane and iron oxide magnetic nanoparticles were integrated into a multifunctional system for melanoma theranostics (MNP@MDSC)^111^. MNP@MDSC camouflaging as an endogenous substance exhibited a prominent tumor tropism and higher antitumor efficacy than MNP@RBC. Particularly, MNP@RBC can serve as a PTT agent to stimulate antitumor immune response via triggering apoptosis of immunogenic cells, reprogramming of tumor-infiltrating macrophages, and suppressing malignant cell metabolic activity. Although the multifunctional system has exhibited good performance in the immune escape, tumor-targeting, MRI, and PTT-induced tumor killing, it is of great potential to simultaneously deliver therapeutic agents for a superior synergistic effect.

### Hybrid cell membrane vectors

Given the important role of RBC membrane retention and hemostatic property of platelets in vivo, PLGA nano-core camouflaged with RBC and platelet hybrid cell membranes was expected to achieve immune evasion and targeted delivery^36^. This eminent technology not just offered constructive instructions to combine the natural properties of various cells through fusing the specific functional membranes, more promoted the application of utilizing nano-vehicles with intricate surface modification chemistry. Most recently, Zhao et al.^112^ produced a fresh type of BNPs by hybridizing membranes derived from platelets and RBC that camouflaged onto pH-sensitive liposomes (PLT-RBC hybrid membrane decoy) to co-deliver a sonosensitizer and a cytotoxic compound for anticancer sonodynamic therapy. With chlorin e6-mediated sonodynamic therapy, tirapazamine-induced chemotherapy, and immunogenic cell death-activated immunotherapy, the unique approach synchronously bestowed the multi-membrane nanoparticle platform with both long-circulation times sourced from RBC and specific targeting to lesion sites from platelets for cascade synergistic therapy of melanoma and metastasis.

In addition to hybridization with platelet membranes, RBC membranes were employed to hybridize with CCM that integrated specific functions from different source cells into a biomimetic nano-system. Along with converting light to heat in BNPs cores, BNPs membranes bestow capability of immune escape and specific accumulation in tumors^37^. When the temperature of tumor sites is higher than that of hyperthermia (42–47°C), the tumor cells can be selectively eliminated, so as to improve the efficacy of PTT^113^. To achieve prolonged circulation time and credible homogenous targeting delivery for melanoma therapy, RBC membranes, and melanoma cell (B16F10 cells) membranes were crossed to encapsulate Dox-loaded hollow copper sulfide nanoparticles (DCuS@[RBC-B16] NPs)^114^. Under 1064 nm NIR laser irradiation, DCuS@[RBC-B16] NPs showed obviously specific self-recognition to the parental cell line while incubating in B16F10 cell line and reinforced homologous-targeting capability in nude mice, resulting in an advantageous synergistic PTT and chemotherapy with complete melanoma suppression. Based on the precious concept of this multifunctional nano-system, combining RBC with homotypic CCM on the surface of corresponding nanoparticles will full of hope for personalized nanomedicine against a variety of cancers^114^. In another research, RBC membranes and MCF-7 cell membranes were fused to cloak melanin nanoparticles (Melanin@RBC-M) to enhance PTT to treat breast cancer^114^. After MCF-7 tumor-bearing athymic nude mice administered intravenously, Melanin@RBC-M in the same membrane protein weight ratios of RBC to MCF-7 displayed observably excellent targeted delivery and superior PTT effect, in comparison with either bulk melanin or different membranes ratios of the BNPs. Aside from the above, targeted delivery of selective CSF-1R inhibitor to tumor-associated macrophages for cancer immunotherapy through RBC-cancer cell hybrid membranes coated pH-responsive copolymer micelle (DH@ECm) was performed^39^. All the results suggested DH@ECm was a potential agent for immune-based intervention targeting strategy in breast cancer. Furthermore, hybrid cell membrane-camouflaged nanoparticles consisting of cancer macrophage–platelet, platelet–leukocyte, and cancer stem cell–platelet were expectedly crossed for different applications including combination therapy and cancer personalized theranostics^115–117^. The vast majority of hybrid membrane-camouflaged nanoparticles emerged as a cross-characteristic of both single membrane-camouflaged nanoparticles, realizing a multifunctional integrated targeted delivery strategy.

## EVs vectors

**Fig. 4:**
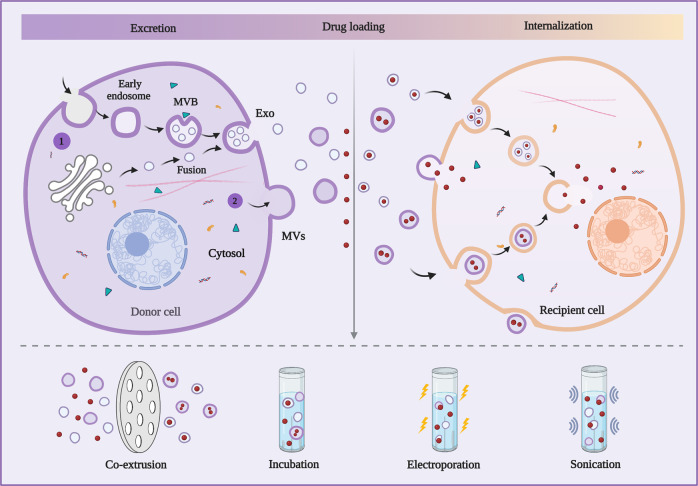
Excretion and internalization of Exo and MVs. (1) Exo is excreted as a result of inward cellular blebbing and fusion of vesicles derived from the Golgi to form multivesicular bodies (MVBs). (2) MVs are formed by plasma membrane blebbing. Exo and MVs are exogenously loaded with therapeutic agents through the main methods including co-extrusion, incubation, electroporation, and sonication. The loading Exo and MVs can be internalized by the recipient cell through exosomal fusion and endocytosis with the therapeutic agents released via membrane fusion or different mechanisms

Compared with microscale cell membrane vectors, EVs vectors exhibit relatively lower loading efficiency limited by their nanoscale size^118^. EVs produced by nearly all cell types in vitro or in vivo are membrane-bound biological nanoparticles that can participate in intercellular communications specifically either in physiological or pathological status^119,120^. Although the subtypes of EVs overlap in terms of the molecular constitution and biological function, EVs are mainly divided into Exo (40–100 nm), MVs (50–1000 nm), and apoptotic bodies (1–5 μm) according to their biogenesis^121^. In any case, the superiority of apoptotic bodies is hindered by their relatively large size, so the leading researchers emphasize the huge role of functionalized Exo and MVs in TDDS^122,123^. EVs are made up of specific molecules such as membrane proteins, lipid bilayers, metabolites, and nucleic acids that reflect their cellular origins^119^. Membrane proteins ensure EVs target specific sites while lipid bilayers protect bioactive contents from the complex body fluid environment and the degradation of extracellular nucleases and proteases^124^. Therefore, EVs can be detached from the source cells and spread through the circulatory system, targeting specific cells or syncretizing with the membranes of distant bound cells to deliver contents (Fig. 4)^125^. Notably, Exo and MVs with low toxicity as the vehicles for brain-targeted delivery have drawn great interest in blood-brain barrier (BBB)-passing nanomedicines while it is infrequent in membrane vectors^126–128^. Since the unprecedented studies on EVs emerging recently, Exo and MVs have been garnering increased attention to serving as vehicles for the targeted delivery of imaging agents and therapeutic molecules (embracing miRNA, siRNA, proteins, peptides, and chemical drugs) due to their preferable physical–chemical properties^25,129,130^. This section summarizes the vectors based on Exo and MVs for imaging and therapeutic strategies.

### Exo vectors

Previous studies have verified the ability of surface-functionalized Exo to image in vivo through multiple methods (i.e., CT, magnetic resonance fluorescence, and bioluminescence) labeling for various disease investigations. Both exogenous and endogenous labeling can be applied to track/image Exo, and the surfaces of Exo are labeled with imaging agents, or parental cells are engineered with genes expressing different biomarkers^131,132^. The surface of Exo was labeled with quantum dots via engineered genes (QDs-labeled Exo) that can be swallowed by cancer cells, demonstrating the possibility of QDs-labeled Exo to act as a contrast agent for tumor-specific labeling^133^. In a separate study, Exo membranes are loaded with superparamagnetic iron oxide nanoparticles and curcumin following conjugation of the neuropilin-1-targeted peptide (RGE-Exo-SPION/Cur) for targeted glioma imaging and therapy^134^. The combination therapeutic strategy of SPION-associated magnetic flow hyperthermia and Cur-associated chemotherapy displays significantly synergistic antitumor effects, which opens up new vistas for accurate imaging and TDDS related to Exo against glioma, even the other cerebral diseases. Besides, significative detection of Exo deep inside the brain is hindered by classic fluorescent and optical imaging^135^. In order to reinforce the efficacy of images in the deep brain, Betzer et al.^136^ created a noninvasive method for CT imaging to tract Exo. Mesenchymal stem cells (MSCs)-derived Exo directly labeled with glucose-coated gold nanoparticles (EGNPs) through an active mechanism connected to GLUT-1 glucose transporter. EGNPs administrated intranasally in acute striatal stroke mice exhibited a more extensive biodistribution, enriched brain accumulation, and longer circulation than the mice administrated intravenously. Moreover, stem cells-derived Exo can be labeled with ultrasmall superparamagnetic iron oxide and MSCs-derived Exo labeled with genetically modified ferritin heavy chain for MRI^137,138^. These inspiring nano-systems based on Exo are supposed to as universal targeted delivery systems to track/image Exo delicately in multifarious pathology, providing information for intervention design.

Natural Exo possesses the capability to extravasate through tumor vessels and passively accumulated to tumor sites, and the engineered Exo are conferred with high specificity and sensitivity towards tumors especially^139,140^. To be exemplified, Exo originated from normal fibroblast-like mesenchymal cells that were engineered to load RNA (iExosomes) targeting oncogenic Kras^G12D^ deemed as a frequent mutation in pancreatic carcinoma^141^. iExosomes undertook the superiorities of extended residence, long circulation, and targeted delivery by contrast with RNA-loaded liposomes, which can be explained by the CD47 on the surface of iExosomes and oncogenic macropinocytosis stimulated by GTPase KRAS^142,143^. Accumulating evidence supported that iExosomes inhibited Kras^G12D^ orthotopic tumor growth and metastasis, as well as extending lifespan in model mice. Arginine-glycine-aspartic acid (RGD)-modified Exo was conjugated with TAT peptides-modified vanadium carbide QDs (V_2_C-TAT@Ex-RGD) for microthermal nucleus-targeted PTT in NIR-II window^144^. The versatile theranostic nanoplatform could substantially eradicate MCF-7 tumors in mice by inducing genetic substance damage and protein denaturation. Priorly, miRNA-loaded Exo was targeted to epidermal growth factor receptor (EGFR)-expressing breast cancer cells^145^. The tumor-targeting capability of Exo was conferred by modified-source cells performing transmembrane region of platelet-derived growth factor receptor integrated to the GE11 peptide. Immature dendritic cells-derived Exo loaded with the potent antitumor drug Dox can target breast cancer cells by fusing Lamp2b and αv integrin-specific iRGD peptide in donor cells^146^. Similarly, Dox-loaded Exo can enhance the therapeutic efficiency of colorectal cancer, breast cancer, and ovarian cancer in mouse models^147,148^.

With the exception of normal cell-derived Exo, it is noteworthy that the Exo secreted by tumor cells inherit homologous surface adhesion molecules from parental cells, also leading to a high tumor specificity in targeted delivery for cancer therapy^149–151^. A programmable-bioactivating prodrug nano-system (EMPCs) based on tumor-derived Exo was conceived for breast cancer anti-metastatic treatment^152^. The reactive oxygen species-responsive thioether-linked PTX-linoleic acid complex and cucurbitacin B were loaded onto polymeric nanoparticles, which were further fabricated with Exo membrane. EMPCs were endowed with multiple functions that can magnify prodrug bioactivation, extend blood circulation, specific target to homotypic cancer cells, strengthen tumor penetration, and inhibit focal metastasis via CTCs elimination and FAK/MMP signaling pathway mediation. A significant antitumor effect was also observed in a recent study that tumor cell-derived Exo was loaded with abundant mRNAs for targeted transcriptional regulation and glioma therapy^153^. mRNA-loading Exo regenerated tumor-suppressor function, reinforced restraint of tumor growth, and prolonged survival time. Besides, tumor-exocytosed Exo conjugated with aggregation-induced emission luminogen (AIEgen) were leveraged to load dexamethasone (DEX) (DEX-Exo@AIEgen), enhancing the efficacy of PDT and alleviate local tissue hypoxia in tumors^154^. Tumor-derived Exo loaded with Dox exhibited the homing ability to donor cells, prolonged retention time in tumor sites, and obvious eradication effect in tumor-bearing mice models^155^.

Brain drug delivery remains a key challenge owing to the restrictive nature of the BBB^156^. Recently, the idea embraced modified Exo as the vehicle for brain-targeted delivery has attracted comprehensive attention in BBB-passing nanomedicines^157–159^. siRNA can be intendedly delivered to mice brain for Alzheimer’s disease by embracing endogenous Exo produced by dendritic cells expressing Lamp2b that can fuse to the neuron-specific RVG peptide^160^. After systemically intravenous treatment in mice, knockdown of BACE1 was found in siRNA-RVG Exo while pre-exposure to RVG Exo did not decrease knockdown in the brain and non-specific uptake in other tissues was not found. Allogenic Exo derived from monocytes and macrophages have a significant advantage to avoid being phagocytosed by the mononuclear phagocyte system^161^. Enlightened by this concept, Haney and his colleagues designed a promising nano-system where Exo was loaded with catalase (ExoCAT) for targeted therapy of Parkinson’s disease^159^. ExoCAT were equipped with favorable loading capacity, control release, and catalase preservation against proteases degradation, especially orientated accumulation in neurons and microglial cells in mice brain, contributing a substantial neuroprotective effect. Similarly, MSCs-derived Exo modified with targeting peptide [c(RGDyK)] was exploited to deliver curcumin for cerebral ischemia therapy^162^. After intravenous injection of cRGD-Exo@Cur, an obvious inhibition of the inflammatory response and cellular apoptosis in the lesion region was confirmed in the transient middle cerebral artery occlusion mice model. Interestingly, Sterzenbach et al.^163^ loaded Exo with exogenous proteins over the mechanism of the evolutionarily conserved late-domain (L-domain) pathway. Exo has loaded in Cre recombinase with a WW tag (WW-Cre Exo) that can be recognized by the L-domain-containing protein Ndfip1 and further generating ubiquitination and successful load. It presented significant aggregation of WW-Cre Exo to recipient neurons in several brain areas, illustrating the important role of engineered Exo to deliver biologically active macromolecule across BBB for brain disorder therapy.

Macrophage-derived Exo was loaded with PTX and then conjugated with aminoethylanisamide-polyethylene glycol (AA-PEG-ExoPTX), in order to enhance the targeted ability for pulmonary metastases through systemic administration^164^. Notably, Exo derived from sorts of cells are modified with the exosomal anchor peptide CP05, which was supposed to bind to an exosomal surface protein CD63 to realize targeted drug delivery^165^. The Exo were assembled into functional drug delivery systems (Exo-CP05) by conjugating with CP05 that connected to M12 (muscle-targeting peptide) and PMO (oligonucleotides drugs), respectively. After intravenous injection in dystrophin-deficient mdx mice, the dystrophin protein-expressing level of Exo-CP05 increased by 18-fold in quadriceps compared with that of CP05-PMO. The groundbreaking design is justified in raising high hopes for fundamental researches and clinical therapeutic transformations in diverse pathophysiological processes. Exo originated from monocyte-derived myeloid cells has been exploited to load curcumin targeting to the inflammatory cells for enhancement of anti-inflammatory activity^166^. In the present study, anti-inflammation therapeutic TPCA-1 was encapsulated in platelet-derived Exo, which specifically targeted pneumonia and calmed down the cytokine storm in the mouse model with acute lung injury finally^167^. The platelet-derived Exo could be applied as a wide nano-system that can intendedly deliver anti-inflammatory agents to diverse inflammatory sites.

### MVs vectors

MVs, also known as shedding MVs or ectosomes, are produced by outward budding of the plasma membrane and distribute widely in size from nanoscale to micron order with nanoscale as the majority^168^. Owing to the capability of delivering biomacromolecule and chemical molecules, MVs have been a sparked intensive interest for therapeutic purpose^160,169,170^. Since 2009, Huang’s laboratory has carried out a series of studies that MVs originated from diverse tumor cells were deemed as significant vectors to deliver payloads, especially chemotherapeutic drugs and oncolytic adenoviruses, for malignant tumors therapy. A549 tumor cells-derived MVs were harnessed as a distinct nanoplatform to deliver oncolytic adenoviruses into the nucleus of tumorigenic cells, which exhibited specifically targeting and potent lethality towards cancer cells in vivo and in vitro^171^. Pre-instillation of tumor cell-derived MVs was conjugated with Dox to enhance the inhibition of orthotopic bladder tumor growth and hematuria occurrence in mice via a lysosomal pathway^172^. In another related study, apoptotic human cancer cells-derived MVs incorporated with MTX (MTX@MVs) alleviate extrahepatic biliary tract obstruction in patients suffered from extrahepatic cholangiocarcinoma (CCA)^173^. MTX@MVs were supposed to destroy CCA cells directly and simultaneously irritate pyroptosis of the cells by a gasdermin E-dependent pathway. The discharge of cellular contents depended on CCA cell death-activated patient-derived macrophages to generate proinflammatory cytokines, evoking neutrophils towards the tumor lesion. A previous study reported that the MVs derived from apoptotic tumor cells could serve as vehicles to deliver chemotherapeutic drug MTX (MTX-MVs)^174^. MTX-MVs demonstrated tumor tropism and strong cellular lethality towards ovarian cancer cells in severe combined immunodeficient mice without obvious side effect. Continuously, the safety and effectiveness studies of MTX-MVs to treat malignant tumors were carried out, which will be discussed in the following section.

Arrestin domain-containing protein 1-mediated MVs (ARMMs) were harnessed to encapsulated diverse bioactive molecules involving tumor-suppressor p53 protein, RNAs, and the genome-editing CRISPR-Cas9/guide RNA complex^175^. These biological molecules-loaded ARMMs showed favorable bio-functional ability inside recipient cells, which can provide a promising nanoplatform to encapsulate and deliver bioactive molecules for diverse biotherapy. Dox and vancomycin were severally loaded onto the MVs derived from RBC, which released efficient encapsulation by supplementing the membrane with additional cholesterol, maintaining the nanostructure, and promoting the retention of a pH gradient^176^. Wan et al.^177^ developed a handy and novel method to obtain drug-loaded MVs for targeted delivery inside MDA-MB-231 mice model. Bone marrow dendritic cell-derived MVs embedded with AS1411 aptamer were loaded with PTX, exhibiting an obvious antitumor effect without systemic toxicity. Comprehending the mechanisms and pathways of these functional MVs delivery systems is not only meaningful for improving the therapeutic efficacy, but also of great significance for facilitating clinical application. Guo et al.^178^ designed proinflammatory macrophage-derived MVs conjugated with Dox (Dox-MiV) for metastatic ovarian cancer therapy and elucidated the modulation mechanism of tumor tropism and drug release. Tumor tropism was achieved by inheritance of CCR2-enriched cell membrane and drug release was attained by SNARE-mediated membrane fusion instead of routine endocytic degradation. In the latest study, hepatocellular carcinoma (HCC)-derived MVs were employed as vectors for sequential nano-catalysts GOD-ESIONs@EVs (GE@MVs) of tumor-specific and cascade nanocatalytic therapy to fight against HCC^179^. The surface of MVs was synchronously loaded with glucose oxidase (GOD) enzyme and extremely small-sized iron oxide nanoparticles (ESIONs) modified with RGD. The theranostic nanoreactor GE@MVs demonstrated a high specific antitumor effect over HCC via catalytic therapeutic modality. Moreover, tumor cell-derived MVs seem to be excellent vehicles for delivering antitumor therapeutics while giving play to stimulate the immune response in vivo^172,180^. Attentionally, most researches have highlighted the tremendous functions of Exo in TDDS, whereas the other subtype EVs—MVs appear to be a relatively new field to explore.

## VNPs vectors

**Fig. 5:**
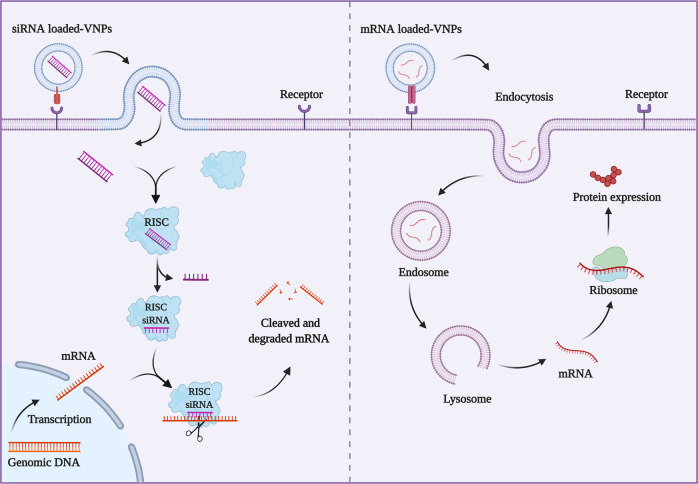
Mechanism of delivering siRNA and mRNA for gene therapy based on VNPs. siRNA or mRNA loaded-VNPs can be internalized into the cytoplasm of the target cells via recognition of glycosylated cell surface receptors on the surface of host cells. siRNA released in the cell cytoplasm will bind to RNA-induced silencing complex (RISC) with the target mRNA and knockdown the specific protein expression in cells. The packaged mRNA is translated into protein after its delivery to the cytoplasm

VNPs are a variety of functional vectors that originated from mammalian viruses, bacteriophages, and plant viruses^181^. Virus-like particles (VLPs), composed of the capsid proteins of the virus, are the nucleic acid-free derivatives of VNPs counterparts without infectious ability in heterologous systems^22^. In terms of mammals, bacteriophages, and plant viruses are relatively safe without the removal of genetic material since these systems are non-integrating and non-replicating, whereas genetic material must be removed from mammalian virus to form VLPs^182^. Unlike lipid consisting cell membrane vectors and EVs vectors, viral vectors consist of proteins, which are also promising carriers for drug delivery owing to their inherent ability to avoid immunological recognition and gain entry into cells to deliver their genes into targeted sites^183,184^. The self-assembly process of viral capsids is formed by protein subunits in different folded forms, which offers a high degree of multivalency in immunostimulatory^184,185^. VNPs with multivalency can also be obtained by functionalization at external, internal, and inter-subunit interfaces for attachment to diverse functional groups provided by protein structures through protein engineering technology or genetic modification^186,187^. However, viral vectors derived from viruses are of potential pathogenicity, so there are still concerns regarding safety and immunogenicity despite the additional efforts to improve their safety profile^184,188^. Owing to the key role of the viral capsid in target and invasion, VNPs are endowed with targeted ability and specific functions by chemical modification or integrating the functional proteins of different viral capsids^184^. Besides, the morphology and size of VNPs not only have an influence on antigen recognition followed by immunological effects but also on the permeability in targeting delivery^189^. After definite modification, VNPs offer comparative superiorities to be suitable carriers that possess distinct morphologies, well-defined nano-size, specific functionalization, good stability, and biocompatibility^22,32^. Since their advantageous biological and physical–chemical properties, VNPs have been proposed as promising vectors to deliver a variety of functional substances embracing contrast agents and therapeutics^190^. In addition to delivering potent chemotherapeutics, VNPs vectors are widely applied for gene therapy as well, especially siRNA and mRNA strategies (Fig. 5). The encapsulation/loading of cargos into VNPs can be prepared by exploiting devise principles such as supramolecular chemistry, chemical attachment, and hierarchical architectures^32^. VNPs vehicles and cargos are prospected to be conjugated tightly through chemical immobilization, non-covalent interactions, and protein engineering^191–193^. Subsequently, the structure can exchange depending on the surrounding environment so as to upload the cargos^192,194^. A wide range of mammalian viral vehicles are exploited for novel delivery strategies, so as to bacteriophages and plant viruses^182,194,195^. This section will focus on a variety of VNPs based on mammalian viruses, bacteriophages, and plant viruses as vehicles to deliver both biomacromolecules and chemical molecules for targeted therapy, as well as presenting imaging briefly.

### VNPs based on mammalian virus

VNPs based on the mammalian virus as vectors for delivering exogenous materials into mammalian cells have been drawn extensive attention in the last few decades^196–198^. Mammalian VNPs are attractive as short circulation and retention time, reducing potential side-effects, and can be modified with peptides, aptamers, or antibodies to load a variety of contrast agents or fluorescent labels for targeted delivery towards specific sites^22,199^. Sun et al.^200^ proposed an original multifunctional nanoplatform derived from Simian virus 40 (SV40-VNPs) for noninvasively targeted image and therapy of atherosclerotic plaques in live ApoE^−/−^ mice. SV40 major capsid protein VP1 was employed as the skeleton to conveniently conjugate three functional components including targeting peptides externally, antykoagulanty Hirulog medially, and NIR QDs internally. SV40-VNPs possessed the capability to selectively deliver Hirulog to atherosclerotic plaques at different stages via conjugation with three types of targeting peptides. The results demonstrated that SV40-VNPs display immense potential to act as individual theranostic nano-systems for biomedicine and biotechnology. To investigate the interaction between virus and host by image formation, Pham et al.^201^ noncovalently attached fluorescent nano-diamonds to a recombinant envelope protein of vaccinia virus (FND-VNPs) for cell or tissue-specific targeting. Targeting was obtained by the conjugation of three typical recombinant capsid proteins (rA27, rDA27, and hybrid of them) to FND. All the FND-VNPs showed specifically targeted binding with glycosaminoglycans, which facilitated imaging and tracking of FND-VNPs in living cells. Owning to their 240 modification sites, hepatitis B core protein (HBc) is considered as a prospective nanoplatform for multifunctional engineering purposes^202,203^. In the hope for image-guided tumor PTT, ICG was creatively encapsulated in RGD-modified HBc VNPs (RGD-VNPs@ICG)^204^. Compared with bulk ICG, RGD-VNPs@ICG exhibited high specificity, long circulation, and aqueous stability, leading to precise imaging and tumor elimination under FL/PA laser irradiation. Although ICG is not preferred as the best therapeutics, the therapeutic effect can be observably optimized via incorporating with RGD-modified HBc VNPs.

Viral vector-mediated gene delivery is an emerging and highly attractive therapeutic solution for the precise treatment of refractory diseases, including infectious disease, cardiac diseases, musculoskeletal diseases, and etc^205^. Martinez-Navio’s study demonstrated that long-term delivery of anti-HIV monoclonal antibodies base on adeno-associated virus (AAV) vectors remained long-lasting antiviral activity, placing great hopes on the prevention and treatment of AIDS^206^. A chronically infected rhesus monkey was functionally cured by continuous delivery of universally effective neutralizing monoclonal antibodies 3BNC117 and 10-1074 from a single dose administration of AAV vector. Ni et al.^207^ constructed a system of atrial-specific in vivo gene delivery using a recombinant AAV (rAAV) driven by the atrial-specific ANF promoter. Intriguingly, AAV with payloads can be ladened with phase-transition microneedles (MNs) for the therapy of ischemic myocardial disease via minimally invasive surgery^208^. MNs fabricated with fluorescent fluorescein isothiocyanate (FITC)-marked AAV (MN-FITC-AAV) and luciferase coding sequence modified-AAV (MN-AAV-LUC) performed excellent targeted delivery and high gene transfection of heart regions. AAV-vascular endothelial growth factor (VEGF)-loaded MNs ameliorated cardiac dysfunction on account of high VEGF expression, functional angiogenesis promotion, and Akt signaling pathway activation. To improve delivery efficiency, Mardry et al.^209^ innovatively designed a rAAV/hydrogel system based on AAV for gene therapy of cartilage defects. The therapeutic rAAV vector, overexpressing chondrogenic sox9 transcription factor in full-thickness chondral defects, was implemented in knee full-thickness chondral defects in minipigs following microfracture. The rAAV/hydrogel system was directly applied to the treated cartilage defects, allowing for in situ gelations. Lentivirus vector is another nanoplatform to deliver nucleic acid for gene editing and therapy. Lu et al.^210,211^ harnessed lentiviral capsid-based BNPs to deliver SaCas9 mRNA and Cas9/sgRNA ribonucleoprotein for transient expression and efficient genome editing. Both systems exhibited lower off-target rates compared with AAV and lentiviral delivery, making it more convenient to deliver specific RNA and ribonucleoprotein for transient Cas9 expression and efficient genome editing. The current study manifests that the genetically and physical–chemical modified viral vectors may further promote the development of gene therapy^205,212^.

More significantly, great efforts have been engaged in modifying other mammalian VNPs as vehicles for the main improvement of antitumor immunity and targeted delivery efficiency^213,214^. HBc was modified genetically to achieve specific recognition and target human EGFR-2 (HER2) expressed by tumor cells^199^. The modified VNPs presented a specific accumulation in HER2-expressing tumor cells in vitro and an obvious accumulation in corresponding tumor-bearing mice models compared with untreated VNPs. In an in-depth study, different regions of HBc were modified with diverse modularized peptides, respectively, and then the modified VNPs were harnessed to encapsulate Dox (denoted as mVNPs-Dox) with non-chemical intervention^215^. It was investigated that VNPs-Dox can target tumor cells specifically by interacting with abundantly expressed integrin α_v_β_3_. In comparison with bulk Dox, mVNPs-Dox showed an excellent ability to suppress tumor growth in B16F10 mice models. Either of the above two studies can provide valuable information on HBc VNPs for modification with peptides or conjugation with cargos, designing more considerable targeted delivery^216^. Analogously, adenovirus dodecahedron (Dd) VNPs were incorporated with either Dox (Dd-Dox) or cap structure analog (cell-impermeant eIF4E inhibitor, Dd-cap)^217^. Both Dd-Dox and Dd-cap had a significant inhibitory effect on the proliferation of different tumor cells, which was equivalent to the effect achieved by dosing five times bulk Dox. In HCC rat models, combination therapy of the two conjugates suppressed tumor growth by 40%, and the level of protooncogenes (eIF4E and c-myc) showed an obvious decrease. Intracellular delivery of the two anticancer agents with different mechanisms demonstrated therapeutic potential against HCC. In addition to decreasing the particle size and modified physic-chemical characteristics of TDDS, creating a desirable system dependent on the tumor microenvironment is also one of the most effective strategies to get over the hurdle of tumor heterogeneity^218–220^. Rabies virus glycoprotein-amplified hierarchical targeted hybrids (RVG-hybrids) were developed to deliver two tumor-penetrating drugs that Dox was loaded on boron-doped graphene quantum dots (B-GQDs@Dox) and palbociclib was loaded on pH-responsive dendrimers (pH-Den@Pb)^221^. The hybrids camouflaged by RVG exhibited significant accumulation in the weakly acidic tumor microenvironment and delivered effectively across BBB to the brain through partial spinal cord transportation. With the high-frequency magnetic field, RVG-hybrids magnetoelectric disassembled into a mass of single B-GQDs and dendrimers, deeply penetrating into tumor location. The synergy of magnetoelectric penetration and chemotherapy significantly inhibited tumor growth and extended the lifespan of orthotopic glioblastoma mice models.

Foot-and-mouth disease (FMD) VNPs are non-enveloped viral particles composed of four virus-encoded structural proteins forming an icosahedral capsid^222^. FMD VNPs involve a highly conserved RGD motif, which can be specifically recognized by integrin receptors highly expressed in several various tumors^223^. Based on previous studies, Yan et al.^224^ primitively constructed FMD VNPs generated in colibacillus to load Dox for targeted drug delivery and potentiate antitumor effect. In nude mice xenograft models, FMD VNPs loaded with Dox remarkably inhibited tumor growth and reduced the pathological damage of Dox caused by off-target delivery. FMD VNPs as delivery vectors were not only harnessed to deliver chemotherapeutics, but also conjugated with adjuvants to enhance immunity and served as target antigens in the meantime. As another application on FMD VNPs, gold-star nanoparticles (AuSNs) were incorporated with FMD VNPs (FMD VNPs-AuSNs) for reinforcing the immunity of the VNPs in FMD^225^. FMD VNPs could access cells more easily in the existence of AuSNs that further activated macrophages and stimulated intensive specific immunoreaction against FMD virus. Zhitnyuk et al.^226^ designed an efficient mRNA delivery system by embracing chimeric VNPs (VSVG-L7Ae VNPs), which was the fusion of protein G of Vesicular stomatitis virus and ribosomal proteins L7Ae of *Archeoglobus fulgidus.* VSVG-L7Ae VNPs can be used as an available system for obtaining high transduction, especially in hard-to-transfect cell lines including mononuclear cells and human-induced pluripotent stem cells.

In addition to the above, employing polyomaviruses as VNPs to deliver bioactive cargos to mammalian cells was also taken into consideration^227^. Dashti et al.^228^ utilized murine polyomavirus (MPyV) VNPs to capture guest proteins by minimal peptide anchors in vivo, and then the cargo-MPyV VNPs can coencapsidate by self-assembling in vitro. MPyV VNPs exhibited substantial uptake into the cytosol of primary human cells. Owing to the encoded affinity for sialic acids extensively existed on the surface of mammalian cells, which can contribute to the delivery of multifunctional proteins to the cytosol. In this direction, the nano-system based on MPyV VNPs envisaged the possibility to serve as the paradigm for capsid proteins self-assembly and cargos self-sorting in co-delivery of multicomponent therapeutics.

### VNPs based on bacteriophage

#### Qβ VNPs

Modified VNPs based on bacteriophages (Qβ, P22, MS2, filamentous bacteriophage, etc.) have proven to be prototypical VNPs platforms for functional purposes in targeted delivery^229–231^. A prominent example is the development of Qβ VNPs engineered with a metalloporphyrin derivant for PDT and a glycan ligand for specific binding of cells with CD22 receptor^232^. On the basis of Qβ VNPs, a photocaged nanoplatform was designed to deliver Dox for the combination of photocaging therapy and chemotherapy^233^. Dual-functionalized VNPs were endowed with improved stability and solubility, exhibiting much less cytotoxicity before photoactivation, highly controlled photo-release, and cellular lethality in breast tumor cells. An updated study manifested a conceived nanoplatform to actively deliver Qβ VNP payloads within ovarian tumor sites^234^. The autonomous propulsion of Qβ VNPs-loaded Mg-micromotors implemented active delivery of complete immunogenicity Qβ VNPs to the peritoneal cavity for immunotherapy of ovarian cancer. Qβ VNPs-loaded Mg-micromotors perfected the intrinsic immunogenicity of Qβ VNP vector and ameliorated tumor immunotherapy in ovarian tumor-bearing mice. To eradicate brain tumors, Pang et al.^235^ constructed the hybrid Qβ VNPs to deliver RNAi via self-assembly and peptides modification methods. Self-assembling fluorescent Qβ VNPs/RNAi hybrids (VNP/RNAi) were co-transformed into *Escherichia coli* (*E. coli*), then the exteriors were modified with dual peptides (dP@Qβ VNP/RNAi). dP@Qβ VNP/RNAi bypassed BBB to deliver RNAi that had an inhibiting effect on DNA reparation, which exhibited synergy with temozolomide in intracranial glioblastomas mice models. In another recent piece of work, an antihypertensive vaccine was designed based on the conjugation of Qβ VNPs and a peptide from Angiotensin II receptor 1 (ATR-Qβ VNPs)^236^. Through a specific dendritic cell functional regulation route over lipid raft reorganization, ATR-Qβ VNPs vaccine stimulated high-titer antibodies aiming at AT1R and decreased systolic blood pressure in hypertensive mice models. Therefore, employing VNPs to develop vaccines and other immunity preparations targeting lipid functional domains is a new field of research with great potential, which is more effective than single ligand–receptor targeting^237^.

Bacteriophages are a multivalent versatile platform that can be modified by combination with different moieties to form synthetic hybrid bacteriophage-based nanocarriers^238^. In previous studies, cell targeting with hybrid Qβ VNPs and engineered PP7 VNPs were applied to display severally epidermal growth factor (EGF) and peptides^239,240^. To realize noninvasive imaging, bacteriophages Qβ and PP7 were successfully hybridized to form a type of hybrid VNPs to load small ultra-red fluorescent protein (smURFP-hVNPs) via a versatile supramolecular disassembly–reassembly approach^241^. Compared with green fluorescent protein (GFP)-loaded analogs, smURFP-hVNPs were bestowed with more sensitive and specific pH response, contributing better imaging performance in the process of phagocytosis in RAW macrophages, and showing different tissue and organ localization.

VNPs have aroused great interest in TDDS as the possibility to enter cells selectively. However, the study on targeting specific cell types in tissues from blood is few and limited^184,194,242^. Several macrolide antibiotics are observed to preferentially accumulate in the lung with more macrophages^243^. Motivated by this rationale, Qβ VNPs were delivered to lung-resident macrophages with the direction of macrolide antibiotics (especially azithromycin) as targeting ligands^244^. Qβ VNPs loaded with azithromycin showed specific tissue tropism to the lung and extended retention time after systemic administration in mice models, which was related to the guidance of azithromycin. Macrolide antibiotics-loaded Qβ VNPs differed from normal TDDS in that the cargo took the responsibility to decide the designated target site, instead of modified the vector to reach the goal. The novel delivery system provided a fresh strategy for intracellular drug delivery for pulmonary infections, even other intracellular infections.

#### P22 VNPs

A previous study proposed that the interior cavity of P22 VNPs were harnessed as vectors for site-specific initiation of atom-transfer radical polymerization with cargo loading, emphasizing the application of multimeric P22 VNPs-polymer conjugates to as MRI contrast agents with the possibility to carry other functional agents^245^. P22 VNPs are deemed as robust supramolecular protein hollow structures with excellent performance on cell type-specific delivery of loaded cargos^246^. Cas9 and a single-guide RNA were encapsulated in truncated P22 VNPs by transfecting SP-Cas9, sgRNA, and the P22-CP plasmids in *E. coli* cells^247^. The RNA-directed endonuclease was functional for sequence-specific cleavage of dsDNA targets, indicating that P22 VNPs were promising vectors to specifically deliver Cas9 to targeted cells. To expand the ability of P22 VNPs to encapsulated guest proteins, the surface of P22 VNPs was modified with trimeric decoration protein, which suggested drastically increasing affinity for B lymphocytes^248^. Besides, P22 VNPs cross-linked with aminoethyl methacrylate were constructed to encapsulate diverse paramagnetic manganese (III) protoporphyrin IX (MnPP) complexes as T_1_-enhanced contrast agents for macromolecular MRI^249^.

#### MS2 VNPs

MS2 capsids are another type of bacteriophage VNPs serving as the effective vehicles to package and targeted deliver chemotherapy drugs, RNA, antibodies, and peptides for medicinal research and development^250–253^. MS2 can be modified precisely through genetic engineering or chemical conjugation, promoting the multivalent display of targeting ligands. Ashley et al.^250^ took advantage of peptide (SP94) modified-MS2 VNPs as the vector to realize cell-specific delivery of various therapeutic agents (including doxorubicin, cisplatin, siRNA cocktails, and protein toxins) against human HCC. Li et al.^254^ produced a recombinant therapeutic vaccine incorporating a 19-nucleotide RNA aptamer (mRNA) and MS2 VNPs (mRNA-VNPs vaccine). The specific exogenous encapsidated mRNA was translated into protein within 12 h after phagocytosed by macrophages in mouse macrophage cell line. mRNA-VNPs vaccine was proved to be a potential intervention to fight against PC, for which it can stimulate extensive humoral and cellular immune responses, obviously inhibiting tumor growth and improving prospects of survival in mice. Apart from this, miRNA (miR-146a) was transported to human peripheral blood mononuclear cells via MS2 VNPs delivery system, which was supposed to suppress genesis and differentiation of osteoclast in precursors^255^. Similarly, novel versatile MS2-chimeric retrovirus-like particles were considered as promising vehicles for efficient delivery of functional RNAs related to osteoblast either in vitro or in vivo^253^. The MS2 VNPs enabled to activate an osteoblast differentiation pathway through the delivery of RUNX2-or DLX5-mRNA into primary human bone marrow MSCs. MS2 VNPs have been extensively employed to act as vehicles for targeted therapeutic agent delivery, yet there is little information about their behavior in vivo. To tap the potential of MS2 VNPs, anti-EGFR antibodies were loaded onto the surface of MS2 VNPs, establishing a delivery nano-system targeted toward receptors overexpressed on breast tumor cells^251^. Bacteriophage can also be modified and engineered directly to deliver nucleic acid without the removal of genetic materials. For example, Namdee et al.^256^ designed a transgene delivery system based on intact filamentous bacteriophages, which were genetically engineered to express RGD and load a mammalian DNA cassette targeted to the gastrointestinal tract.

### VNPs based on plant virus

#### TMV-based VNPs

Among numerous plant viruses, TMV VNPs are undergoing development to implement targeted delivery and molecular imaging in the biomedical field. Natural TMV possesses a preferable aspect ratio (AR), and especially performs desirable biodistribution and tumor specificity, making it suitable as targeted drug delivery vectors^257–259^. Recently, Hu et al.^260^ constructed polydopamine-modified TMV to load T_1_-MRI contrast agent gadolinium for simultaneously implementing photoacoustic/magnetic resonance bimodal imaging and PTT to kill PC cells. A previous study took advantage of cavity structure and high-AR nanotubes composed by capsid protein of TMV to encapsulate cationic photodynamic agents for PDT over melanoma^261^. The TMV-PS was investigated with the ability to deliver the cargo to melanoma cells specifically. Phenanthriplatin (PhenPt), a cationic monofunctional Pt(II) compound, exhibits a distinct tumor cell profile compared to traditional platinum therapeutics^262^. To investigate its anticancer effect, Czapar et al.^263^ utilized TMV as a delivery system to encapsulate PhenPt (PhenPt-TMV) via a one-step loading protocol driven by electrostatic interactions For fluorescence imaging, TMV was marked with sulfo-Cy5 dye at exterior tyrosine side chains followed by the encapsulation of PhenPt. Compared with PhenPt, cisplatin, and TMV, PhenPt-TMV group showed obvious small tumors and longer survival than control groups in MDA-MB-231 xenografts athymic mice models. The fluorescence imaging indicated that PhenPt-TMV accumulated in tumor sites with significantly high intensity, and meanwhile reached nontarget organs including kidneys, liver, and lungs. Besides, the TDDS based on TMV was designed to load cisplatin for tumor cell inhibition. TMV was modified with carbohydrates as targeting ligands and conjugated with mannose or lactose on the exterior surface (Cisplatin@TMV-Man and Cisplatin@TMV-Lac)^264^. Cisplatin@TMV-Man and Cisplatin@TMV-Lac exhibited obvious superiority of endocytosis and apoptosis efficiency in galectin-rich MCF-7 cell line and ASGPR-overexpressing HepG2 cell line, respectively. Gao et al.^265^ decorated TMV capsid protein with a small molecular fluorous ponytail to form spherical nanoparticles induced by fluorous interaction, and cisplatin was loaded onto the modified TMV via metal-ligand coordination. Cisplatin@F-TMV enhanced the tumor-cytotoxic effect in Hela cells, and the release of cisplatin was investigated to depend on the low pH of the tumor microenvironment and cancer cell lysosomes. TMV was proposed as the vehicle to deliver cisplatin to platinum-resistant (PR) ovarian tumor cells^266^. The delivery system showed not only a highly targeted efficacy towards PR ovarian tumor cells but also the potential to restore efficacy in PR disease.

Apart from the fluorous interaction mentioned above, native TMV rods can be reshaped into spherical nanoparticles tailored by a bottom-up RNA-templated self-assembly approach and induced by a thermal cycler^265,267,268^. The biotin-modified TMV spherical nanoparticles and TMV rods were harnessed to conjugate or encapsulate Dox for efficient payload delivery to breast cancer^190^. Dox-conjugated TMV nanoparticles showed a superior inhibited efficacy over Dox-encapsulated TMV rods in MCF-7 and MDA-MB-231 cell lines. Analogously, Dox was bio-conjugated to a self-assembling nanoscale disk that TMV capsid proteins were recombinantly expressed by a double arginine mutant^269^. To improve its solubility and bioavailability, Dox-TMV disks were modified with PEG (PEGylated-TMV@Dox). PEGylated-TMV@Dox had significant cytotoxicity in accord with free Dox in GBM cells, but unmodified Dox-TMV remained nearly no cytotoxicity. Motivated by the prominent gene transfection efficiency of VNPs, Tian et al.^270^ designed a rod-like gene-silencing vehicle based on TMV. The exterior surface of TMV was modified with transacting activator of transduction (TAT) peptide, which was subsequently loaded with silencer GFP siRNA (siRNA@TMV-TAT). The existence of TAT enhanced cell internalization and obtained endo/lysosomal escape. siRNA@TMV-TAT knocked down the GFP-positive cells to a much lower level in GFP-expressing mouse epidermal stem cells (ESCs/GFP) in vitro. Furthermore, the high GFP downregulating capacity of siRNA@TMV-TAT was demonstrated in mice bared high GFP-expressing metastatic HCC (MHCC97-H/GFP) tumors subcutaneously. Accumulating evidence supports that TMV nanoparticles offer great advantages as diverse functional vectors to deliver payloads for therapeutic strategies including chemotherapy, PDT, gene therapy, etc.

TMV is a remarkably versatile vector to deliver contrast agents for various imaging techniques of abnormalities sites such as atherosclerotic plaques and tumors^271,272^. VCAM-1 expresses highly and has a major role in atherosclerosis development, which stresses the availability of VCAM-1 as an attractive target for atherosclerosis imaging^273^. A VCAM-1-targeted probe based on TMV (VCAM-1-TMV) was proposed for dual MRI and optical imaging in atherosclerotic plaques^272^. TMV rod was conjugated with VCAM-1 targeting peptides externally, and labeled with contrast agents internally, in which chelated gadolinium ions for MRI and Cy5 NIR dye for optical imaging. Injected with VCAM-1-TMV, a remarkable specificity to VCAM-1 and high accumulation was observed in atherosclerotic ApoE^−/−^ mice models. S100A9 (myeloid-related protein 14), is another biomarker to indicate vulnerable plaques in rupture-prone atherosclerotic lesions for diagnosis of atherosclerosis with high-risk factors^274^. In a similarly practical example, Park et al.^275^ modified TMV with S100A9-targeting peptides externally and Cy5 molecules internally for imaging and diagnosis of atherosclerosis in ApoE^−/−^ mice models. Both VCAM-1 and S100A9-targeted TMV nano-system exhibited a distinct tropism to the plaque, indicating the tendency to apply TMV-based VNPs for imaging and the possibility to deliver drugs for the treatment of atherosclerosis. Towards this direction, the engineered TMV VNPs (Dy-Cy7.5-TMV-DGEA) were prepared to enhance SNR in ultra-high-field MRI and NIR imaging of PC^276^. The internal cavity of TMV was decorated with dual contrast agents (Dy^3+^ and Cy7.5), followed by an external conjugation of an Asp-Gly-Glu-Ala (DGEA) peptide targeting PC-3 cells with a high expression of integrin α_2_β_1_. In vitro and in vivo studies elucidated that Dy-Cy7.5-TMV-DGEA was a credible candidate for dual model imaging for diagnosis and prognosis of PC. Moreover, TMV was fabricated with BF3-NCS dye for intravital two-photon imaging in the mouse brain vasculature^277^.

#### Cowpea mosaic virus (CPMV)-based VNPs

CPMV is 30 nm-sized icosahedral particle, which possesses a large surface area to volume ratio allowing modification with multifunctional groups^278^. In previous studies, CPMV was decorated with functional molecules such as targeting ligands and chemical compounds for delivery to specific cells^279,280^. For example, CPMV modified with alkyne-functionalized carboxyl dendrons was harnessed to incorporate with Zn-EpPor PS (PS-CPMV) for dual delivery to macrophages and cancer cells^281^. It has been demonstrated that the specificity of CPMV for the immunosuppressive subpopulation of macrophages and targeting ability to tumor cells^282,283^. Therefore, Lam et al.^284^ utilized CPMV as the vector to load mitoxantrone (MTO-CPMV) to evaluate the cytotoxicity towards U87 MG cells. After the successful preparation of MTO-CPMV, Oregon Green 488 was conjugated to the VNPs to carry out cell uptake studies. With the combination therapy of tumor necrosis factor-related apoptosis-inducing ligand, MTO-CPMV showed a more potent in vitro antitumor efficacy compared with either solo therapy. CPMV offers an attractive alternative to cancer imaging and therapy tools to synchronously present targeting moieties, imaging agents, or therapeutics for maximum therapy and diagnosis efficacy^285^. On the basis of another plant virus known as cowpea chlorotic mottle virus (CCMV), Lam’s laboratory proved that the delivery of siRNA therapeutics could realize a more efficient gene knockdown^286^. siRNA targeting GFP or the forkhead box transcription factor (FOXA1) was loaded onto CCMV. Whereafter, the cell-penetrating peptide (M-lycotoxin peptide L17E) was attached to siRNA-CCMV to further improve cell uptake and intracellular trafficking. Hu et al.^287^ designed an integrated NIR fluoresce and MRI bimodal contrast agent based on capsid protein of Physalis mottle virus (PhMV) for targeted PC imaging. PhMV nanoparticles were not only labeled with contrast agents Cy5.5 and Gd^3+^ complexes internally, but also conjugated with mPEG and targeting peptides DGEA externally. In PC-3 tumor-bearing mice models, the NIR fluoresce signal and MRI obviously highlighted the tumor site and distinguished it from the surrounding tissues for an extended long time. The bimodal imaging system provided a highly intriguing nanotechnology platform for the targeted delivery of contrast agents and even drugs simultaneously.

## Clinical trials

Till now, EVs have not been applied extensively since a handful of clinical trials have either been undertaken or are currently ongoing. In these clinical trials, Exo produced by different cell types is functionally modified as effective vectors for targeted delivery in chemotherapy or biotherapy. Phase I clinical trial from 2011 investigated the ability of plant Exo to deliver curcumin to colon tumors and normal colon tissue (NCT01294072). The effect of exosomally delivered curcumin on the immune modulation, cellular metabolism, and phospholipid profile of normal and malignant colon cells in subjects who are undergoing surgery for newly diagnosed colon cancer are characterized. On the promise of safety and feasibility, tumor antigen (IFN-γ-)-loaded dendritic cell-derived Exo serving as maintenance immunotherapy after first-line chemotherapy target to non-small-cell lung cancer (NCT01159288). This Phase II trial verified the ability of dendritic cell-derived Exo to facilitate the NK cell arm of antitumor immunity in patients with advanced non-small-cell lung cancer. In 2019, a Phase I/II study aimed to assay the administration of allogenic MSC-derived Exo loaded with miR-124 in the treatment of disabled patients with acute ischemic stroke (NCT03384433). In another example, loading of MSC-derived Exo with Kras^G12D^ siRNA was expected to prevent the deterioration of patients with metastatic pancreas cancer (NCT03608631). Most timely, Codiak Biosciences announced that a Phase I/II study was carried out, where the first-in-human study of CDK-002 (exoSTING) in subjects with advanced/metastatic, recurrent, injectable solid tumors, emphasizing squamous cell carcinoma of the head and neck, triple-negative breast cancer, anaplastic thyroid carcinoma, and cutaneous squamous cell carcinoma (NCT04592484). Codiak’s other potential product exoIL-12 was on a Phase I study in London (http://www.codiakbio.com/therapeutics/). The aim of the clinical trial was to assess the safety tolerability, pharmacokinetics, and pharmacodynamics of exoIL-12 after a single incremental subcutaneous injection in healthy volunteers, and then to apply the determined dose of exoIL-12 to patients with stage IA-IIB cutaneous T-cell lymphoma. In addition, the effects of RBC units-derived Exo on platelet function and blood coagulation and plasma-derived Exo on cutaneous wound healing were evaluated in early phase clinical trials (NCT02594345, NCT02565264).

Tumor cell-derived MVs encapsulating chemotherapeutic drugs might be an effective remedy to cope with refractory malignant tumors. Based on tumor cell-derived MVs, Huang and his fellows conducted a sequence of clinical trials in the field of solid tumors and complications such as malignant pleural effusion (MPE), malignant ascites, CCA, and lung cancer, providing a brand-new solution for the treatment of malignant tumors. A Phase II clinical trials from 2013 recruited 30 malignant ascites or pleural effusion patients to evaluate the safety and effectiveness of tumor cell-derived MVs (NCT01854866). After a week, administration of cispatin-loaded MVs, >95% cancer cells in the malignant fluids were killed in advanced lung cancer patients while intrathoracic injection of free cisplatin did not display an antitumor effect in the other three patients^180^. In cispatin-loaded MVs-treated patients, not merely the tumor cells in the malignant fluids were diminished, but also the liquid volume, color, and clarity of malignant fluids as well as symptoms of patients were obviously improved. Owing to the effectivity and low toxicity of tumor cell-derived MVs@MTX in mice models, Guo et al.^288^ carried out a further study of intrapleural injection of a single dose of autologous tumor cell-derived MVs@MTX to evaluate their safety, immunogenicity, and clinical activity in 11 end-stage lung cancer patients with MPE (NCT02657460). Delightedly, the MVs@MTX exhibited the targeting ability to malignant cells and immunosuppression to microenvironment without adverse side-effect. A new treatment measure clinical study demonstrated that tumor cell-derived MVs@MTX served as a potent immunotherapeutic drug for patients with MPE (ChiCTR-ICR-15006304). The MVs@MTX specifically targeted MPE by mobilizing and activating neutrophils via a pleural catheter triggered neutrophils gather in patients through macrophage-secreted CXCL1 and CXCL2^289^. In 2015, a Phase I clinical trial was conducted to access the safety and efficacy of tumor cell-derived MVs@MTX tubed by PTCD/ENBD drainage for advanced bile duct cancer to bile duct perfusion prospective, single-arm, multicenter clinical study (ChiCTR-OIB-15007589). The latest research proved that tumor cell-derived MVs@MTX could distinctly alleviate biliary obstruction in patients with CCA^173^. Further progress in the specific mechanisms of EVs biogenesis, transport, cargo delivery, and function are remained to be manifested for significant clinical translation.

AAV gene delivery system is ushering in a new and exciting era in the field of biotherapy worldwide. In the last ten years, viral vector-based gene delivery was approved for the treatment-refractory diseases, such as lipoprotein lipase deficiency (Glybera), leber congenital amaurosis (Luxturna), spinal muscular atrophy (Zolgensma), acute lymphoblastic leukemia (Kymriah), and large B-cell lymphoma (Yescarta)^290–292^. Clinical trials utilizing viral vector-based therapies are increasingly implemented in patients suffering from genetic and non-genetic diseases, and long-term therapeutic effects have been achieved for these refractory diseases. In 2017, Luxturna was approved by FDA for the treatment of RPE65 mutation-associated retinal dystrophies, which was the first subretinal administration product based on rAAV vector. Subsequently, clinical trials of AAV2-hRPE65v2 gene therapy (Luxturna) are carried out to access whether Luxturna is safe and effective in different individuals with leber congenital amaurosis (NCT03602820, NCT03597399, NCT00516477, NCT00999609, NCT01208389). Besides, the spinal muscular atrophy type 1 (SMA1) participants treated with a single intravenous infusion of AAV vector containing gene coding (Zolgensma) for survival motor neuron (SMN), exhibiting extended lifespan, superior performance of motor milestones, and better motor function than in historical cohorts (NCT02122952)^293^. Subsequent clinical trial aimed to evaluate the safety and tolerability of ocular AAV-RS1 vector (AAV8-scRS/IRBPhRS) gene transfer to the retina of participants affected with X-linked juvenile retinoschisis (NCT02317887). Preliminary data from Phase III study showed rapid motor function improvements in SMA1 patients, paralleling Phase I results^294^. The continuous clinical trials will evaluate its long-term safety and efficacy in the subjects with SMA1 (NCT03421977, NCT03306277). Furthermore, a Phase I, randomized, blinded, dose-escalation study of rAAV1-PG9DP rAAV vector coding for PG9 antibody was launched in healthy male adults (NCT01937455). Intramuscular administration of rAAV1­PG9DP appeared safe and well tolerance among small groups of healthy men for immunoprophylaxis to prevent HIV^295^.

Except for AAV vectors, the vectors based on retrovirus, alphavirus, and adenovirus were also translated to fight against human diseases. Rexin-G, the first targeted gene therapy strategy, which was a tumor-targeted retrovector bearing a cytocidal cyclin G1 construct for the treatment of osteosarcoma, sarcoma, pancreatic cancer (NCT00505271, NCT00572130, NCT00505713) A γ retroviral vector received marketing authorization for ex vivo gene therapy of adenosine deaminase severe combined immune deficiency and Phase I/II studies were investigated on recessive dystrophic epidermolysis bullosa (NCT02984085)^296^. Similarly, in a child with junctional epidermolysis bullosa, the human epidermis can be regenerated entirely by autologous transgenic keratinocyte cultures via retroviral vectors^297^. Another clinical study indicated that γ retroviral vector showed epigenetic suppression of transgenic T-cell receptor expression through the vector methylation in adoptive cell transfer therapy^298^. Moreover, the anticancer vaccine based on an alphaviral vector enhanced HER2-specific memory CD8^+^ T cells and exhibited obvious inhibition of breast cancer in clinical testing^299^. At present, SARS-CoV-2 vaccine candidates based on adenovirus have been emerging in Phase I/II clinical trials (NCT04341389, NCT04400838). The previous and new researches and clinical trials offered promising approaches that the other stem cell-mediated combined ex vivo cell and gene therapies can be achieved through the novel idea. The clinical trials of BNPs based on EVs and VNPs are summarized in Table 2.

**Table 2:** Clinical trials of BNPs for human diseases

Vectors	Therapeutic agents	Clinical indications	Phase	Identifiers
Plant Exo	Curcumin	Colon cancer	I	NCT01294072
Dendritic cell-derived Exo	Tumor antigen	Non-small cell lung cancer	II	NCT01159288
Allogenic MSC-derived Exo	miR-124	Acute ischemic stroke	I/II	NCT03384433
MSC-derived Exo	Kras^G12D^ siRNA	Metastatic pancreas cancer	I	NCT03608631
Exo	Stimulator of interferon genes agonist	Advanced solid tumor	I/II	NCT04592484
Tumor cell-derived MVs	Cispatin	Malignant ascites or pleural effusion	II	NCT01854866
Tumor cell-derived MVs	MTX	MPE	II	NCT02657460
Tumor cell-derived MVs	MTX	MPE	-	ChiCTR-ICR-15006304
Tumor cell-derived MVs	MTX	Advanced bile duct cancer	I	ChiCTR-OIB-15007589
AAV	SMN	SMA1	I	NCT02122952
AAV	Ocular gene	X-linked juvenile retinoschisis	I/II	NCT02317887
AAV	SMN	SMA1	III	NCT03421977, NCT03306277
AAV	Gene coding for PG9 antibody	Immuneprophylaxis	I	NCT01937455
Retrovirus	Cytocidal cyclin G1 construct	Osteosarcoma, sarcoma, pancreatic cancer	I/II	NCT00505271, NCT00572130, NCT00505713
γ retrovirus	COL7A1 Complementary DNA	Recessive dystrophic epidermolysis bullosa	I/II	NCT02984085
Adenovirus	Gene coding for spike protein of SARS-CoV-2	COVID-19	II	NCT04341389
Adenovirus	Gene coding for surface spike protein	COVID-19	II/III	NCT04400838
Oncolytic adenovirus	TMZ-CD40L and 4-1BBL	Pancreatic cancer	I/II	NCT02705196

## Clinical challenges

The biological vectors inspired by cell membranes, EVs, and viruses inherit intrinsic characteristics including immune escape and tumor tropism from the source cells and viruses. Theoretically, these vectors can realize targeting delivery, long-circulation, and appropriate biocompatibility with low side-effects through diverse functional modifications^300^. Since the vectors are complicated entities generated by organisms, the translation of BNPs from laboratory to clinical use is impeded with different obstacles than for synthetic nanoparticles. The design and utilization of almost all vectors to deliver payloads are still in the laboratory stage owing to various technical and practical limitations^22,301^. Existing challenges of these vectors for clinical application mainly contain three aspects: stability, large-scale production, and safety & effectiveness.

First, one of the concerns is the instability of BNPs. Size, shape, components, physical–chemical characteristics of vectors vary from modifications and different sources, which can significantly affect their performance^302^. Tumor cell-originated MVs can deliver Dox with high efficiency via downregulating cytospin-A to alter the physical softness of MVs^303^. 3D-MVs presented more obvious tumor tropism, easier extravasation, and deeper tumor penetration, as well as more efficient internalization by tumor cells and side population cells in contrast with 2D-MVs. The cell sources for vectors produce still remain uncertain, which may restrict delivery efficacy owing to their high heterogeneity^153,304^. VNPs expressed by heterologous systems may integrate genome materials leading to adverse side-effects in the host cell^305^. In basic researches, the vectors derived from a single type of resource can be endowed with limited function while hybrid vectors can be conferred with multi-functions without elaborated modification, indicating the hybrid vectors will be the focus in the future^117,240^. BNPs are secularly unstable because they are difficult to be preserved and easily to be deactivated, which hampers large-scale production of the nanoparticles^306^. Since the vectors originated from diverse sources differ in their stability, the stability of BNPs should be observed^32^.

Second, the complex preparation methods are difficult to achieve large-scale production, which limits the clinical translation of BNPs. A more intelligent and effective manner for extraction and coating is urgently required to obtain biocompatible vectors with high purity, plasticity, and reproducibility^26,32,37^. On the one hand, manufacturing techniques are still in the primary stage relating to immature isolation, purification methods, low yields, improper modification, as well as insufficient loading and delivery efficiency with therapeutic payloads^307^. Various purification methods including filtration, ultracentrifugation, and PEG precipitation could have an adverse influence on the quality and quantity of finished products^308^. To yield multifunctional nanoparticles, specific membrane decorations will inevitably be demanded, which may raise the risk of unfavorable side-effects^309^. For instance, excessive immune cell membrane-coated nanoparticles may facilitate inflammation response viab interaction with the immune system, thus causing the release of the pathological mediator^88^. In terms of CCM-derived vectors, the nuclear and genetic material in tumor cells is supposed to be completely removed to eliminate carcinogenic risk^27^. On the other hand, knowledge about BNPs is hampered by the methodology accessible to the efficient combination of biological vectors and synthetic particles. Hence, considerable approaches to yield more BNPs are demanded before BNPs can be applied reliably in a therapeutic setting^304^. Towards this direction, some endeavors have been dedicated to addressing these issues. Beyond conventional bulk electroporation (BEP), a cellular nanoporation biochip was exploited to achieve the large-scale generation of functional mRNA-encapsulating Exo, which was supposed to stimulate diverse source cells to produce Exo with nucleotide sequences^153^. The clinical trials based on BEP to produce Exo have emerged as it is mentioned in the last section. In comparison with CEP and other Exo-production technologies, the biochip released up to 50-folds more Exo and 1000-folds more transcripts of exosomal mRNA. When taking practical use into consideration, the laboratory protocols are generally not scalable and adaptation to industry standards is required^22^.

Third, the biological safety and bioactive effect of BNPs remain unclear in human beings^309^. Translating these tissue-specific, non-toxic, and non-immunogenic delivery technologies into clinical practice requires comprehensive in vivo studies to estimate the possible side effects and therapeutic effects. The mechanism behind the vectors’ benefits also warrants further investigation in humans, in order to turn potential vectors into clinical translation^310^. Unlike small-molecule drugs that have certain evaluation criteria: Lipinski’s Rule of Five^311^, there is currently no corresponding criteria to evaluate whether BNPs are feasible and accessible to develop into biopharmaceuticals. Despite the lack of criteria, it is universally acknowledged that the potential of BNPs to be exploited into drugs can be preliminarily judged from their physicochemical, biochemical, pharmacodynamic, pharmacokinetic properties^312^. Besides the evaluation of efficacy, it is of great significance to consider the combination of predictive toxicological safety evaluation supported by cogitative investigation on different perspectives including absorption, distribution, metabolism, excretion, and toxicokinetic performance^313^. Owing to the efficacy and safety closely associated with the biomimetic nano-vectors, the design and investigation of BNPs need to address the important physicochemical properties of the nano-vector that may contribute to hazards when the BNPs are applied in humans, promoting the clinical translation of TDDS based on BNPs.

## Conclusion and perspectives

**Fig. 6:**
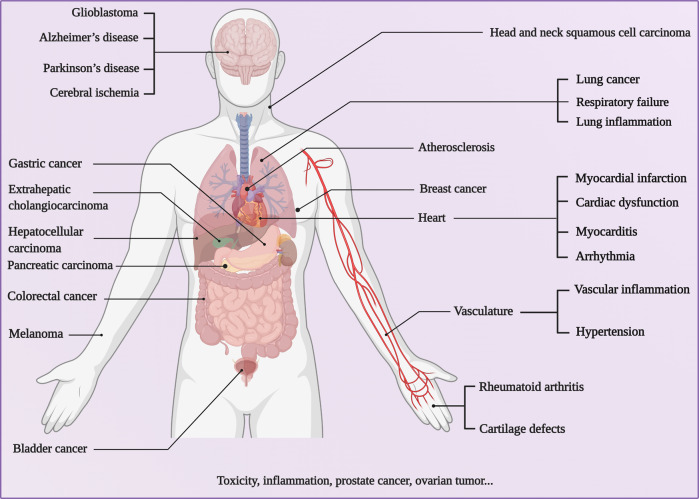
BNPs ameliorate various diseases via targeted delivery of therapeutic agents

This review summarizes the state-of-the-art of BNPs based on cell membranes, EVs, and viruses, as well as highlighted the relevant biomedical applications (Table 3 and Fig. 6). The multifarious strategies based on plenty of biomimetic vectors have already been proposed to intendedly deliver therapeutic and imaging agents. The specific functions and properties including specific targeting, immune invasion, and long circulation are integrated into one nanoplatform via modification on both vectors and synthetic inner cores. With the combination therapy and synergistic action, BNPs achieve potent therapeutic effects over refractory diseases, especially malignant tumors. Although fundamental studies demonstrated these BNPs are quite perspective, issues concerning the toxicity, biodistribution, and immunology of various types of nano-vectors need to be fully investigated. Continued study of BNPs biology and comprehensive understanding of the correlative therapeutic challenges and limitations will lay the solid foundation for future clinical success.

**Table 3:** Diverse vectors for targeted delivery highlighted in this review

Vectors	Synthetic inner cores	Payloads	Targeting mechanisms/sites	Indications	References
RBC membrane	MSNs	Dox	Laser-activated tumor-specific accumulation	Breast cancer	^43^
	PLGA	RAP	EPR effect in atherosclerotic lesions	Atherosclerosis	^18^
	Nanocrystals	DTX	Tumor-targeting ligands	Glioma	^47^
	Cationic liposomes	AmB	Specific affinity between pathogenic fungi and erythrocytes	Fungal infection	^53^
CCM	Aluminum phosphate nanoparticles	CpG	Lymph node-resident APCs	Melanoma	^14^
	Calcium pyrophosphate nanoparticles	-	Targeting to dendritic cells	Melanoma	^75^
	Porous Au@Rh	ICG	Homologous targeting	Breast cancer	^78^
	PLGA	ICG	Homologous targeting	Breast cancer	^79^
	Au nanostars	Dox	Homologous targeting	Melanoma	^81^
	Boron nitride nanotubes	Dox	Homologous targeting	Glioblastoma	^83^
	MSNs	Dox	Homologous targeting	Prostate cancer	^85^
	Gelatin nanoparticles	Cisplatin	Homologous targeting	Head and neck squamous cell carcinoma	^86^
Neutrophil membrane	Polycaprolactonepoly(ethylene glycol) nanoparticles	Sparfloxacin	Inflammatory targeting	Inflammation	^15^
	PLGA	-	Binding to immunoregulatory molecules	Rheumatoid arthritis	^87^
	Cationic liposome	MTX	Targeting to macrophages	Inflamed skeletal muscle; ischemic heart	^92^
	Poly(latic-co-glycolic acid) nanoparticles	Carfilzomib	CTC- and niche-targeting	Cancer metastasis	^98^
NK cell membrane	Liposomes	Dox	Targeting tumor cells with the help of NK cell markers	Breast cancer	^23^
	mPEG-PLGA nanoparticles	TCPP	Binding of tumor-targeting proteins	Primary and abscopal tumor	^88^
	Carboxylate terminated PLGA	Gd lipid	Tumor-homing natural killer cell membrane	Tumor imaging	^16^
Macrophage membrane	UCNPs	-	Cell-cell adhesion for cancer targeting	Breast cancer	^103^
	Liposome	Emtansine	α_4_β_1_ integrin-VCAM-1 interactions	Lung metastasis of breast cancer	^89^
	Hollow bismuth selenide nanoparticles	Quercetin	CCL2/CCR2- mediated recruitment	Lung metastasis of breast cancer	^106^
	PEGylation nanoparticle	PTX	IGF1R targeting ligand	Breast cancer	^17^
Platelet membrane	DSPE-PEG polymers	Gevokizumab	Infarct-homing ability of platelets	Acute myocardial infarction	^110^
	Porous metal-organic framework nanoparticles	siRNA	Tumor-targeting capability of platelets	Breast cancer	^19^
Myeloid-derived suppressor cell membrane	Iron oxide magnetic nanoparticles	-	MDSC with high affinity to melanoma	Melanoma	^111^
Platelet-RBC hybrid membrane	Liposome	Chlorin e6 and tirapazamine	Biomimetic surface molecules	Melanoma and lung metastasis	^112^
RBC-cancer hybrid membrane	CuS nanoparticles	Dox	Homologous targeting	Melanoma	^114^
RBC-cancer hybrid membrane	-	Melanin nanoparticles	Homologous targeting	Breast cancer	^317^
Macrophage-derive Exo	-	SPION and Cur	Neuropilin-1-targeted peptide	Glioma	^134^
MSCs-derived Exo	EGNPs	-	Binding of glucose and GLUT-1 glucose transporter	CT imaging of acute striatal stroke	^136^
Normal fibroblast-like mesenchymal cell-derived Exo	-	siRNA or shRNA	RNA payloads specific to oncogenic Kras^G12D^	Pancreatic cancer	^141^
Exo	PEGylated nanoparticles	V_2_C QDs	RGD-targeting tumor cells and TAT peptides targeting nucleus	Breast cancer	^144^
Tumor-derived Exo	PEG-PCL NPs	PTX-S-LA and CuB	High affinity between CCM and homotypic Exo membrane	Breast cancer metastasis	^152^
Tumor cell-derived Exo	-	mRNAs	Targeting peptides	Glioma	^153^
Dendritic cell-derived Exo	-	siRNA	Lamp2b fused to the neuron-specific RVG peptide	Alzheimer’s disease	^160^
Macrophages-derived Exo	-	Catalase	Surface adhesion proteins	Parkinson’s disease	^159^
Exo	-	Biologically active proteins	Floxed reporter cells	Brain diseases	^163^
Exo	-	Oligonucleotides drugs	Muscle-targeting peptide	Dystrophin deficiency	^165^
Tumor cell-derived MVs	-	MTX	Attracting neutrophils	CCA	^173^
DCs-derived MVs	-	PTX	Aptamer AS1411 targeting nucleolin	Breast cancer	^177^
CCM-derived MVs	Extremely small-sized iron oxide nanoparticles	Glucose oxidase	Homologous and RGD-targeting	Hepatocellular carcinoma	^179^
SV40-VNPs	-	QDs and Hirulog	Targeting peptides	Atherosclerotic plaques	^200^
HBc VNPs	-	ICG	RGD-targeting tumor cells	Glioblastoma	^204^
AAV	-	VEGF	Targeting to myocardium	Ischemic myocardial disease	^208^
HBc VNPs	-	Dox	Integrin α_v_β_3_-mediated targeting	Glioblastoma	^215^
Dd VNPs	-	Dox or cap analog	Targeting liver cancer cells	Hepatocellular carcinoma	^217^
RVG-hybrids VNPs	QDs and pH-responsive dendrimers	Dox and Pb	RVG-guided and hierarchical targeting	Orthotopic brain tumor	^221^
FMD VNPs	-	Dox	RGD-targeting tumor cells	Carcinoma of the uterine cervix	^224^
Qβ VNPs	-	RNAi	Peptide targeting to tumor cells	Glioblastoma	^235^
Qβ VNPs	-	Azithromycin	Ligands targeting lung-resident macrophages	Pulmonary infections	^244^
P22 VNPs	-	Cas9 and a single-guide RNA	Targeting to specific cell	Genetic diseases	^247^
MS2 VNPs	-	mRNA	Inducing specific immunity	Prostate cancer	^254^
TMV VNPs	-	PhenPt	Tumor-homing ability of AR engineering	Triple-negative breast cancer	^263^
TMV VNPs	-	Silencer GFP siRNA	Passive targeting	Hepatoma	^270^
CPMVVNPs	-	MTO	Targeting to cerebral endothelial cells	Glioblastoma	^284^
